# VILIP3 attenuates neuronal apoptosis and oxidative stress via Nrf2 activation in the pathogenesis of Alzheimer’s disease

**DOI:** 10.1186/s10020-025-01280-9

**Published:** 2025-06-10

**Authors:** Shasha Huangfu, Xiaoyu Sang, Shiyue Zhou, Haixia Liu, Dongqing Cui, Yansheng Du, Xinyue Xing, Wenyan Liu, Jianzhong Bi, Zhaohong Xie

**Affiliations:** 1 Department of Neurology, The Second Hospital, Cheeloo College of Medicine, Shandong University, Jinan, 250033 China; 2 Department of Neurology, Weihai Municipal Hospital, Cheeloo College of Medicine, Shandong University, Weihai, 264200 China; 3 Department of Ultrasound, Shandong Provincial Hospital Affiliated to Shandong First Medical University, Jinan, 250021 China

**Keywords:** Alzheimer’s disease, VILIP3, Oxidative stress, Apoptosis, Nrf2, Neuroprotection

## Abstract

**Background:**

Alzheimer’s disease (AD) is a common neurodegenerative condition characterized by amyloid-β protein (Aβ) deposition, which is central to its pathological changes. Oxidative stress also plays an important role in its pathogenesis. Visinin-like protein 3 (VILIP3), a neuronal calcium sensor protein, is abnormally expressed in the brains of patients with AD; however, the exact mechanism remains unclear. This study investigated the role of abnormal VILIP3 expression in AD pathogenesis and its underlying mechanisms.

**Methods:**

We used 5×FAD mice as an in vivo model of AD and Aβ_1−42_-treated SH-SY5Y cells to construct an in vitro model. Changes in VILIP3 expression were assessed in both models. VILIP3 was overexpressed in the hippocampus of 5×FAD mice and SH-SY5Y cells using adeno-associated virus (AAV) or plasmid transfection. Cognitive function, Aβ deposition, neuronal damage, synaptic plasticity, apoptosis, oxidative stress, and other relevant indices were evaluated.

**Results:**

VILIP3 was expressed at lower levels in AD model mice and cells than in controls. Overexpression of VILIP3 ameliorated cognitive deficits, reduced Aβ deposition and neuronal loss in 5×FAD mice, and attenuated oxidative stress levels and apoptosis in 5×FAD mice and Aβ_1−42_-treated SH-SY5Y cells. Furthermore, VILIP3 activated nuclear factor E2-related factor 2 (Nrf2) and increased the expression of downstream antioxidant genes. The amelioration of apoptosis and oxidative stress by VILIP3 was blocked by Nrf2-specific inhibitors.

**Conclusions:**

VILIP3 mitigates oxidative stress and apoptosis by activating the Nrf2 signaling pathway, thereby alleviating neuropathological damage and cognitive dysfunction in AD.

**Supplementary Information:**

The online version contains supplementary material available at 10.1186/s10020-025-01280-9.

## Background

Alzheimer’s disease (AD), as a major public health challenge in the 21st century, is characterized by progressive cognitive decline (Scheltens et al. 2021), with the core molecular pathological hallmarks of the disease being the formation of neurofibrillary tangles by aberrant phosphorylation of tau proteins and senile plaques made up of Aβ deposits (Hyman et al. 2012). AD has a complex and unclear etiology and pathogenesis, and no effective treatment currently exists in clinical practice (Abeysinghe et al. 2020). Thus, an in-depth exploration of AD’s etiology and pathogenesis is crucial to developing effective preventive and curative measures.

Currently, the mainstream hypotheses for the pathogenesis of AD include the amyloid cascade hypothesis, the tau protein hypothesis, the inflammation hypothesis, and the oxidative stress hypothesis. Oxidative stress is a key link that connects these pathways (Bai et al. 2022). An imbalance in the production and removal of reactive oxygen species (ROS), a key mediator of oxidative stress (Jomova et al. 2023), leads to DNA/protein/lipid damage and exacerbates AD pathology by inducing neuronal death via the mitochondrial apoptotic pathway (Cai et al. 2022; Schieber et al. 2014). Excess ROS directly affects synaptic activity and neurotransmission, leading to cognitive dysfunction (Tönnies et al. 2017). Oxidative stress has also been reported to increase the expression and activity of beta-site amyloid precursor protein cleaving enzyme 1 (BACE1) and γ-secretase, which promotes the formation of Aβ (Hernández-Rodrìguez et al. 2020; Tamagno et al. 2008). Subsequently, Aβ aggregation further increases oxidative stress, creating a vicious cycle (Liu et al. 2023).

Nuclear factor E2-related factor 2 (Nrf2) is a transcription factor that regulates the cytoprotective system and controls the transcription of NADPH quinone oxidoreductase 1 (NQO1), heme oxygenase-1 (HO-1), components of the glutathione family, including glutamate cysteine ligase (GCL), and glutathione synthetase (GSS) (Murakami et al. 2023), play a key role in maintaining cellular redox homeostasis and responding to oxidative stress. Decreased expression of Nrf2 and its driver genes has been observed in the brains of older individuals and patients with AD. Numerous studies have shown that Nrf2 dysfunction is involved in the pathogenesis of AD, including Aβ and p-tau deposition (George et al. 2022; Guan et al. 2022). Therefore, Nrf2 is considered a significant therapeutic target for AD, and activating Nrf2 is a potential strategy for neuroprotection (Osama et al. 2020). Nrf2 activators tested in various clinical trials have shown positive effects in in vivo models of AD (Bourdakou et al. 2023).

Visinin-like protein 3 (VILIP3) is a member of the visinin-like protein (VSNL)-a subfamily of neuronal Ca^2+^-sensor (NCS) proteins, which also includes VILIP-1, VILIP-2, hippocalcin, and neurocalcin δ (Braunewell 2012). VILIP3 was found to interact with microsomal cytochrome b5 and alter the interaction between NADH-cytochrome b5 reductase and cytochrome b5 in a Ca^2+^-dependent manner (Oikawa et al. 2004, 2016). Cytochrome b5 reductase is part of the plasma membrane redox system, which is impaired in AD (Hyun et al. 2010). In non-small cell lung carcinoma, the knockdown of VILIP3 increased the oxygen consumption rate and ROS levels in H1299 cells (Wang et al. 2022a, b). These previous results suggest that VILIP3 may be involved in the maintenance of redox homeostasis in organisms and is associated with oxidative stress. In recent years, VILIP3 has been found to regulate the growth and proliferation of tumor tissues and cross-talk with several signaling pathways (Chen et al. 2022; Tan et al. 2023; Zhang et al. 2016, 2019). Previous studies have shown that VILIP3 can inhibit the activity of Glycogen Synthase Kinase 3β (GSK3β) by promoting the phosphorylation of GSK3β at the Ser9 site (Zhang et al. 2016, 2019). GSK3β is a negative regulator of Nrf2 (Hayes et al. 2015; Wang et al. 2022a, b). It is suggested that VILIP3 may be involved in the oxidative stress response process by activating the Nrf2 pathway. VILIP3 is widespread in the brain (Bernstein et al. 1999), and previous studies have shown that the neuronal expression levels of VILIP3 are reduced in the cerebral cortex of patients with AD and that the abnormal location of VILIP3 correlates with typical neuropathological features of AD (Braunewell et al. 2001). Genome-wide association studies have shown that genetic variation in VILIP3 is associated with late-onset AD (Braunewell 2012). However, the specific role of VILIP3 in AD progression and the regulatory mechanism remains unclear.

In this study, we focused on the changes in VILIP3 expression in the AD models, as well as the biological functions and mechanisms related to VILIP3 in AD disease progression through a combination of in vivo and in vitro experiments. We demonstrated that VILIP3 attenuates oxidative stress damage and reduces apoptosis by activating the Nrf2 signaling pathway, which further ameliorates neuropathological damage and cognitive impairment in AD.

## Methods

### Cell culture and drug treatment

Human neuroblastoma cells (SH-SY5Y) were provided by the Cell Bank of the Chinese Academy of Sciences (Shanghai, China). Cells were cultured in high-glucose Dulbecco’s Modified Eagle Medium (Gibco, California, USA) with 1% penicillin-streptomycin (Solarbio, Beijing, China) and 10% fetal bovine serum (Lonsera), and incubated at 37 °C with 5% CO2. Aβ_1−42_ oligomer (Qiangyao Biotechnology, Shanghai, China) was used to establish an in vitro model of AD, and ML385 (MCE, USA) was used as an inhibitor of Nrf2.

### Cell transfection

PcDNA3.1-VILIP3 and pcDNA3.1-empty plasmids (Jipeng Biotechnology, Shandong, China) were used to overexpress VILIP3 or to establish controls in cells. Briefly, SH-SY5Y cells were inoculated in 6-well plates and transfected according to the instructions of Lipofectamine 3000 (Invitrogen, Carlsbad, USA). The sequence of PcDNA3.1-VILIP3 is listed in Table S1.

### Cell viability

Cell viability was measured using the Cell Counting Kit (CCK)−8 assay (MCE, USA). SH-SY5Y cells were seeded in 96-well plates with a density of 1 × 10^4 cells/well (100µL/well) and subsequently subjected to different treatments based on experimental groups. At the end of the intervention, 10 µL of CCK8 reagent was added to each well and incubated at 37 °C for 2 h. The optical density at 450 nm was measured using a microplate reader (Biotek, Winooski, Vermont, USA).

### Cell apoptosis

Cell apoptosis was detected using an Annexin V-FITC/PI Apoptosis Detection Kit (Vazyme, Nanjing, China). Briefly, SH-SY5Y cells were seeded in 6-well plates and collected at the end of the intervention. 1× binding buffer containing Annexin V-FITC and Propidium Iodide (PI) was then added according to the manufacturer’s instructions and kept in the dark for 10 min. Apoptotic cells were detected using flow cytometry (Beckman).

### Animals

Male 5×FAD and wild-type mice, both on the C57BL/6 J background, were obtained from Beijing Hua-fu-kang Biotechnology Co., Ltd. 5×FAD mice are transgenic model mice with 5 familial AD mutations (Oakley et al. 2006). All animal experiments were conducted in accordance with the National Institutes of Health Guide for the Care and Use of Laboratory Animals and were reviewed and approved by the Ethics Committee of the Second Hospital of Shandong University (approval number: KYLL2024754).

### Stereotactic AAV injection

AAV-GFP-VILIP3 and its blank vector (AAV-GFP-Ctrl) (GeneChem, Shanghai, China) were used to overexpress VILIP3 in an in vivo model of AD or to establish the control group. Wild-type mice (henceforth referred to as C57 mice) and 5×FAD mice were reared until 6 months of age for hippocampal stereotaxic injection. After the mice were anesthetized with pentobarbital sodium (40 mg/kg, i.p.), they were fixed to a brain stereotaxic device (Stoelting, USA) and the skull was exposed. The hippocampus was located on the surface of the skull, marked, and a small hole was drilled at the injection site. C57 mice were injected with AAV-GFP-Ctrl, and 5×FAD mice were randomly divided into two groups injected with AAV-GFP-VILIP3 or AAV-GFP-Ctrl, respectively. The virus was injected into the left and right hippocampus of the mice at a rate of 0.2 µL/min using a 2 µL microsyringe (Gaoge, Beijing, China). After completing the virus injection, the mouse scalp was sutured. The Morris water maze (MWM) test was performed 4 weeks later. The sequence of AAV-GFP-VILIP3 is listed in Table S1.

### MWM test

The MWM test is commonly used in laboratories to test the spatial learning and memory abilities of rats and mice (D’Hooge et al. 2001). The MWM (ScanBio, Nanjing, China) is a circular pool with a diameter of 120 cm in which the water temperature is maintained at 22 ± 2 °C. The swimming area was divided into four equal quadrants and a white platform with a diameter of 9 cm was placed 1 cm underwater at the center of one quadrants to allow the mice to swim up and stand on it. During the place navigation period, the mice enter the water from different quadrants each day, and software is used to record the time it takes for the mice to find the platform from the time they enter the water. If the mice did not find the platform within 60 s, it was guided to swim to the platform and allowed to remain there for 20 s. This period will continue for 5 days. During the spatial probe period, the platform was removed the day after the last training session. Mice were allowed to enter the water at the furthest position from the initial platform position, and the software was used to record their movement trajectories for subsequent analysis.

### Hematoxylin-eosin (HE) staining and Nissl staining

After the MWM test, the mice were anesthetized and then perfused transcardially with saline and 4% paraformaldehyde. Subsequently, brains were collected and fixed in 4% paraformaldehyde for 24 h. The dehydrated brains were embedded in paraffin, and paraffin brain slices with a thickness of 4 μm were prepared.

The slices were stained for HE staining experiments using an HE Staining Kit (Servicebio, Wuhan, China) according to the manufacturer’s instructions. Nissl staining experiments of the slices were also performed using a Nissl Staining Kit (Servicebio, Wuhan, China). Staining results were scanned using a digital slide scanner (NanoZoomer S60, Hamamatsu, Japan).

### Immunohistochemistry (IHC)

IHC experiments were performed using the Mouse Ultrasensitive Two-Step Immunohistochemical Detection Kit (zsgb-bio, Beijing, China). Previously prepared paraffin brain slices were sequentially de-paraffinized and rehydrated using xylene and ethanol in different concentration gradients, followed by an antigen retrieval step according to the protocol recommended by the primary antibody manufacturer. Slices were then incubated with an endogenous peroxidase blocker for 10 min at room temperature, followed by incubation with Aβ antibody (Biolegend, USA) conjugated with 5% goat serum at 4 °C overnight. The following day, a reaction-enhancing solution was added and incubated for 20 min at 37 °C, followed by an HRP-conjugated secondary antibody, which was incubated for 20 min at 37 °C. Diaminobenzidine (DAB) chromogenic solution was then added and incubated for 5 min at room temperature, and hematoxylin was added and incubated for 20 s. The slices were then dehydrated and sealed, and images were captured using the digital slide scanner mentioned above.

### ROS assay

SH-SY5Y cells were seeded in 12-well plates and cultured according to the experimental design. At the end of the culture, positive and negative control wells were prepared according to the instructions of the ROS Fluorometric Assay Kit (Elabscience, Wuhan, China), and the cells were incubated with dichlorofluorescein diacetate (DCFH-DA) working solution for 60 min at 37 °C in the dark. The ROS levels were detected via flow cytometry, as mentioned above, or using a fluorescence microscope (Olympus IX73, Japan).

A tissue single-cell suspension was prepared according to the manufacturer’s instructions. Briefly, mice were anesthetized and subjected to transcardial saline perfusion, after which the brain tissue was removed, and freshly isolated hippocampal tissue was immediately placed in pre-cooled phosphate-buffered saline (PBS). The hippocampal tissues were cut into pieces, enzyme digestion solution was added, and the cells were digested at 37 °C for 20 min, during which they were shaken intermittently. The digestion was stopped by adding a medium containing serum. The cell suspension was filtered through a 70-µm cell strainer (BIOFIL, Guangzhou, China) and washed twice with PBS, followed by dihydroethidium (DHE) incubation and ROS levels measurement according to the ROS Fluorometric Assay Kit (Elabscience, Wuhan, China) instructions. ROS levels were detected via the previously mentioned flow cytometry.

### Oxidative stress assay

Malondialdehyde (MDA), total superoxide dismutase (T-SOD), and reduced glutathione (GSH) levels in mouse hippocampal tissue and SH-SY5Y cells were determined using the MDA assay kit (Nanjing Jiancheng, Nanjing, China), T-SOD assay kit (Nanjing Jiancheng, Nanjing, China), and Reduced Glutathione Colorimetric Assay Kit (Elabscience, Wuhan, China). The tissue and cell samples were processed according to the manufacturer’s protocols. A Bicinchoninic acid (BCA) Protein Assay Kit (Solarbio, Beijing, China) was used to measure the protein content. The absorbance value was measured using a microplate reader, as mentioned above.

### Real-time quantitative polymerase chain reaction (RT-qPCR)

Total RNA samples were obtained from SH-SY5Y cells and mouse hippocampal tissues using an RNA-Quick Purification Kit (ES Science Biotech, Shanghai). DNA was removed from the RNA samples, and the RNA was transcribed into cDNA using an Evo M-MLV RT Kit (Accurate Biotechnology, Hunan, China). The cDNA was mixed with SYBR qPCR Master Mix (Vazyme, Nanjing, China) and specific primers, and RT-qPCR was performed on a QuantStudio 5 real-time PCR system (Thermo Fisher Scientific, Marsiling, Singapore). All Experimental procedures were performed according to the manufacturer’s instructions. Primer sequences are listed in Table S2.

### Western blot (WB)

Whole-cell proteins were extracted from mouse hippocampal tissues and SH-SY5Y cells using a mixed lysate of radio-immunoprecipitation assay (RIPA) lysis buffer (Beyotime) and phenylmethanesulfonyl fluoride (PMSF) (Beyotime, Beijing, China). As directed by the manufacturer’s instructions, nuclear proteins were isolated using the Nuclear and Cytoplasmic Protein Extraction Kit (Beyotime, Beijng, China). Proteins were quantified using the BCA Protein Assay Kit. The extracted proteins were then heated at 95 °C for 10 min with 5×loading buffer (Beyotime, Beijing, China). Equal amounts of the protein samples and protein ladder (26616, Thermo Fisher Scientific, USA) were loaded onto sodium dodecyl-sulfate polyacrylamide gels. Proteins were then transferred to polyvinylidene fluoride (PVDF) membranes (Merck Millipore, Darmstadt, Germany) and then blocked with 5% non-fat powdered milk solution for 1.5 h at room temperature. The membranes were then incubated with the antibody overnight at 4 °C. Details about the antibodies used are provided in Table S3. The following day, membranes were incubated with HRP-labelled goat anti-rabbit immunoglobulin G (IgG) (H + L) (zsgb-bio, Beijing, China) for 1 h at room temperature. After washing with TBST (Yamei, Shanghai, China), the images were visualized using an enhanced chemiluminescence (ECL) reagent (Millipore, Ireland) and captured using a chemiluminescence imager (Tanon4800, Shanghai, China).

### Immunofluorescence (IF)

SH-SY5Y cells were seeded in a 15-mm glass substrate with a density of 1 × 10^4 cells/well, and treated according to different experimental groups after adhesion. The cells were then fixed using Immunol Staining Fix Solution (Beyotime, Beijing, China) for 10 min at room temperature, followed by the addition of 0.5% TritonX-100 (Solarbio, Beijing, China) for 20 min at room temperature, and then, 5% goat serum (Beyotime, Beijing, China) was added and incubated at room temperature for 1 h. The cells were incubated with the appropriate amount of primary antibody and at 4 °C overnight. The following day, the secondary antibody (goat anti-rabbit IgG Alexa Fluor Plus 488, Abcam) was added and incubated at room temperature for 1 h. DAPI (Sigma, USA) was used to stain the nuclei, and the fluorescent images were captured using a laser scanning confocal microscope (Zeiss, LSM 800, Germany).

Paraffin brain slices were de-paraffinized and rehydrated and the antigen was retrieved as previously described. Then, 0.5% TritonX-100 was added and incubated for 10 min at room temperature, followed by 5% goat serum, which was incubated for 1 h at room temperature. Primary antibodies were then added and incubated at 4 °C overnight. Subsequently, the secondary antibody (goat anti-rabbit IgG Alexa Fluor Plus 488, goat anti-rabbit IgG Alexa Fluor Plus 647, Abcam) was added and incubated for 1 h at room temperature. DAPI staining solution was used to stain the nuclei, and fluorescent images were captured using the laser scanning confocal microscope.

### Statistical analysis

Statistical analyses were performed using GraphPad Prism 9.5 software, and all data are presented as the mean ± standard deviation (SD). Two-group comparisons were analyzed by Student’s t-test. Multi-group data were analyzed by one-way ANOVA, and behavioral test data were analyzed by two-way ANOVA, followed by Tukey’s post-hoc test. Statistical significance was set at *p* < 0.05.

## Results

### VILIP3 is minimally expressed in 5×FAD mice and Aβ_1−42_ oligomer-induced SH-SY5Y cells

**Fig. 1:**
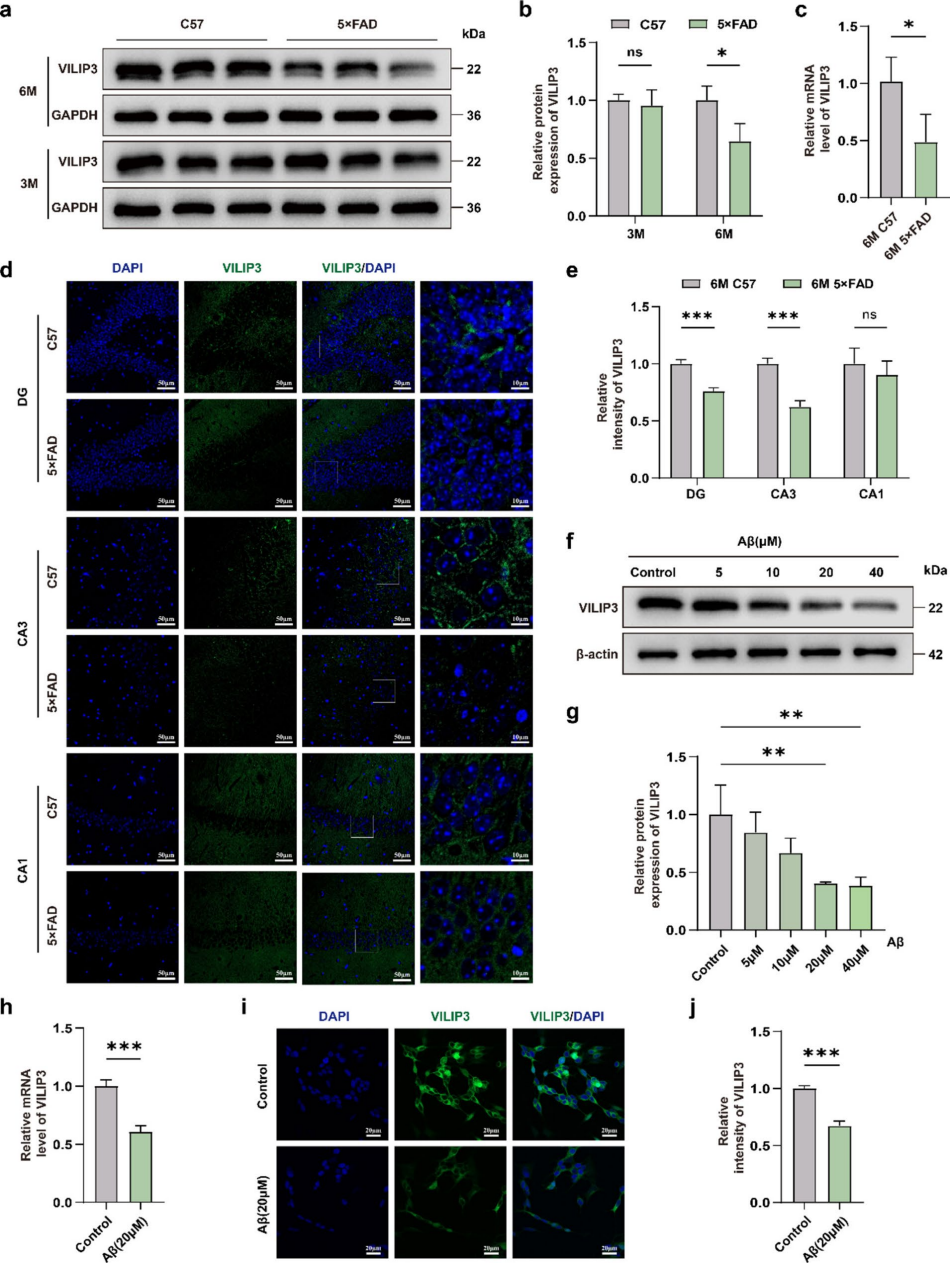
Expression of VILIP3 in in vivo and in vitro AD models.** a**,** b** Relative expression of VILIP3 in the hippocampus of 3-month-old and 6-month-old C57 and 5×FAD mice as determined using WB (*n* = 3). **c** Relative VILIP3 mRNA levels in the hippocampus of 6-month-old C57 and 5×FAD mice determined by RT-qPCR (*n* = 3). **d**,** e** Relative intensity of VILIP3 in three regions (DG, CA3, and CA1) of the hippocampus of 6-month-old C57 and 5×FAD mice as determined using IF (*n* = 3). The first figure on the right (Scale bars, 10 μm) is an enlargement of the second figure on the right (Scale bars, 50 μm). **f**,** g** Relative expression of VILIP3 in SH-SY5Y cells induced by different concentrations of Aβ_1−42_ as determined using WB (*n* = 3). **h** VILIP3 mRNA levels in control and 20 µM Aβ_1−42_-induced SH-SY5Y cells as determined using RT-qPCR (*n* = 3). **i**,** j** Relative density of VILIP3 in control and 20 µM Aβ_1−42_-induced SH-SY5Y cells as determined using IF (Scale bars, 20 μm) (*n* = 3). Data are presented as means ± SD. *P < 0.05, ** *p* < 0.01 and *** *p* < 0.001; ns, no significance. 3 M, 3-month-old; 6 M, 6-month-old

To investigate the effect of VILIP3 in AD, we first observed the protein expression levels of VILIP3 in the hippocampus of C57 mice and 5×FAD mice at different ages. Our results showed that VILIP3 levels were not significantly changed in 3-month-old 5×FAD mice, but were significantly lower in 6-month-old 5×FAD mice, than in C57 mice of the same age (Fig. 1a, b). We also assessed the mRNA levels of VILIP3 in the hippocampus of 6-month-old 5×FAD and C57 mice. Our results showed that the mRNA levels of VILIP3 were significantly reduced in 5×FAD mice, consistent with the results of WB (Fig. 1c). To validate the expression and distribution of VILIP3 further, we performed IF experiments, which showed that VILIP3 levels were significantly reduced in 5×FAD mice compared to those in C57 mice in the DG and CA3 regions, but no such difference was observed in the CA1 region (Fig. 1d, e).

We then used Aβ_1−42_ to treat SH-SY5Y cells to establish an AD model in vitro. We exposed SH-SY5Y cells to different concentrations of Aβ_1−42_ for 48 h and observed that VILIP3 levels were reduced in a dose-dependent manner, with statistical significance at 20µM (Fig. 1f, g). Similarly, the mRNA levels of VILIP3 were significantly lower in the Aβ-treated group than in the control group when SH-SY5Y cells were treated with Aβ_1−42_ at 20 µM (Fig. 1h). Results from IF experiments also showed reduced VILIP3 expression in the Aβ-treated group (Fig. 1i, j). Therefore, our findings suggest that VILIP3 is expressed at low levels in AD models in vivo and in vitro.

### VILIP3 ameliorates cognitive impairment in 5×FAD mice

**Fig. 2:**
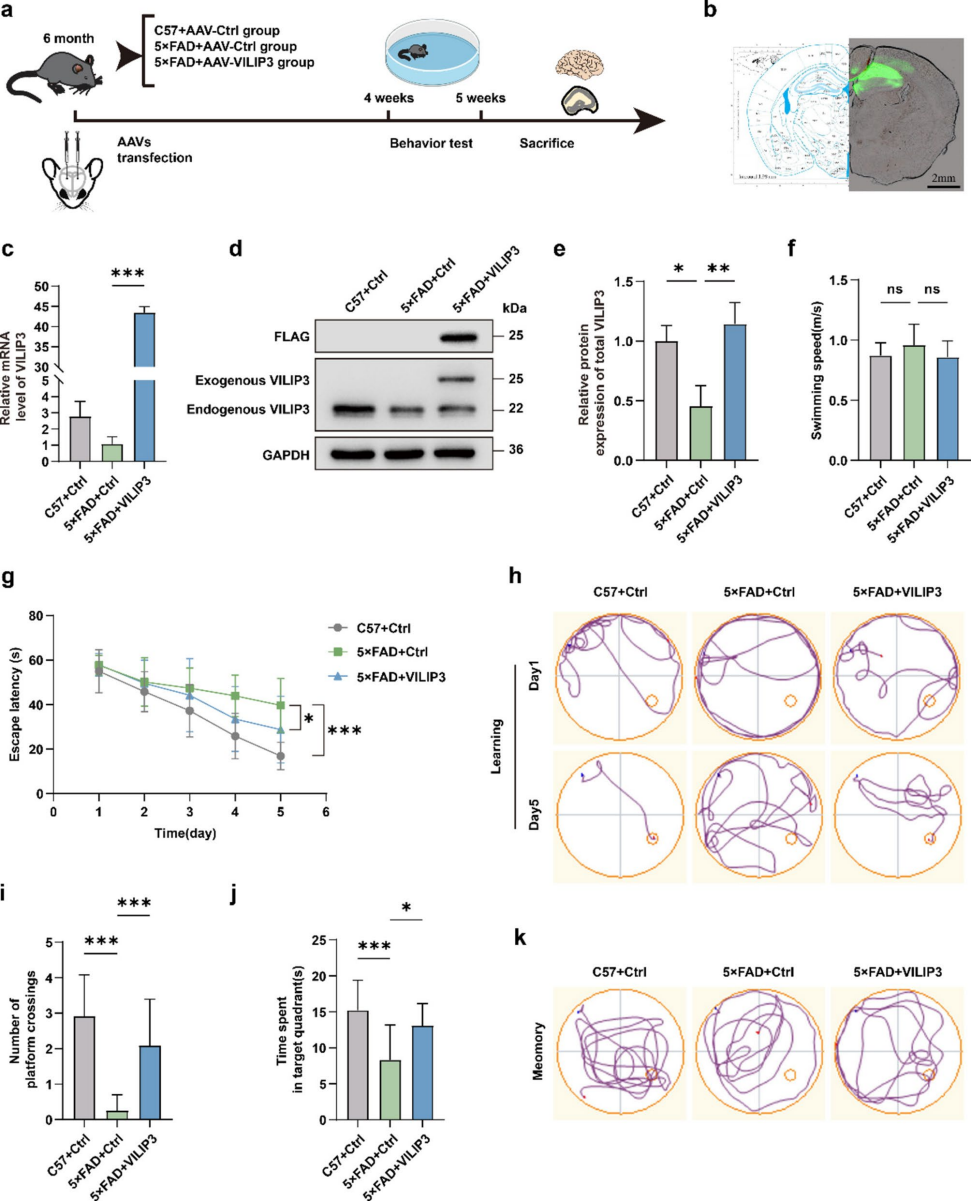
Effects of VILIP3 overexpression on cognitive function in 5×FAD mice.** a** Schematic diagram of experimental design. 6-month-old C57 mice were transfected with AAV-GFP-Ctrl (i.e., C57-Ctrl group), and 6-month-old 5×FAD mice were transfected with AAV-GFP-Ctrl (i.e., 5×FAD-Ctrl group) or AAV-GFP-VILIP3 (i.e., 5×FAD-VILIP3 group), behavioral tests were performed 4 weeks after transfection, and the mice were euthanized after the behavioral test for subsequent experiments. **b** Spontaneous green fluorescence in the hippocampus (Scale bars, 2 mm). **c** Relative VILIP3 mRNA levels in the hippocampus of mice in the C57 + Ctrl, 5×FAD + Ctrl, and 5×FAD + VILIP3 groups, as determined via RT-qPCR (*n* = 3). **d**,** e** Relative VILIP3 protein levels in the hippocampus of mice in the C57 + Ctrl, 5×FAD + Ctrl, and 5×FAD + VILIP3 groups, as determined via WB (*n* = 3). **f** Swimming speed in the MWM test (*n* = 12). **g** Mean escape latency in the MWM test (*n* = 12). **h** Representative movement trajectories on the first day and last day of the MWM place navigation period (*n* = 12). **i** Number of platform crossings during the MWM spatial probe period (*n* = 12). **j** Time spent in the target quadrant during the MWM spatial probe period (*n* = 12). **k** Representative movement trajectories during the MWM spatial probe period. Data are presented as means ± SD. *p < 0.05, **p < 0.01, and ***p < 0.001; ns, no significance

To observe the effect of VILIP3 on cognitive function and its regulatory mechanism in mice, we constructed an AD mouse model overexpressing VILIP3 in the hippocampus using hippocampal stereotactic injection and AAV transfection (Fig. 2a). We randomly divided 6-month-old 5×FAD mice into two groups, while C57 mice of the same age were used as the control group. 5 weeks after injection, we observed spontaneous green fluorescence of AAV-GFP in the mice hippocampus, indicating the correct location of the injection (Fig. 2b). The results of RT-qPCR showed that we successfully upregulated VILIP3 expression in the hippocampus of 5×FAD mice at the gene level (Fig. 2c), and WB and IF results indicated that the protein expression level of VILIP3 was also successfully upregulated (Fig. 2d, e and Fig. S1a, b). 4 weeks after injection, we performed MWM behavioral experiments to determine the spatial learning and memory abilities of the mice. There was no significant difference in the swimming speed of the mice among the groups (Fig. 2f). During the place navigation period, the escape latency of the mice gradually decreased. On the 5 th day of training, the escape latency of the 5×FAD-Ctrl group was significantly longer than that of the C57-Ctrl group, whereas the escape latency of the 5×FAD-VILIP3 group was shorter than that of the 5×FAD-Ctrl group (Fig. 2g, h). During the spatial probe period, the platform was removed, and the 5×FAD-Ctrl group exhibited a significantly lower number of platform crossings and less time spent in the target quadrant than the C57-Ctrl group. However, these metrics were improved in the 5×FAD-VILIP3 group compared to those in the 5×FAD-Ctrl group (Fig. 2i-k). These results suggest that overexpression of VILIP3 ameliorates cognitive deficits in 5×FAD mice.

### VILIP3 ameliorates neuropathological damage in 5×FAD mice

**Fig. 3:**
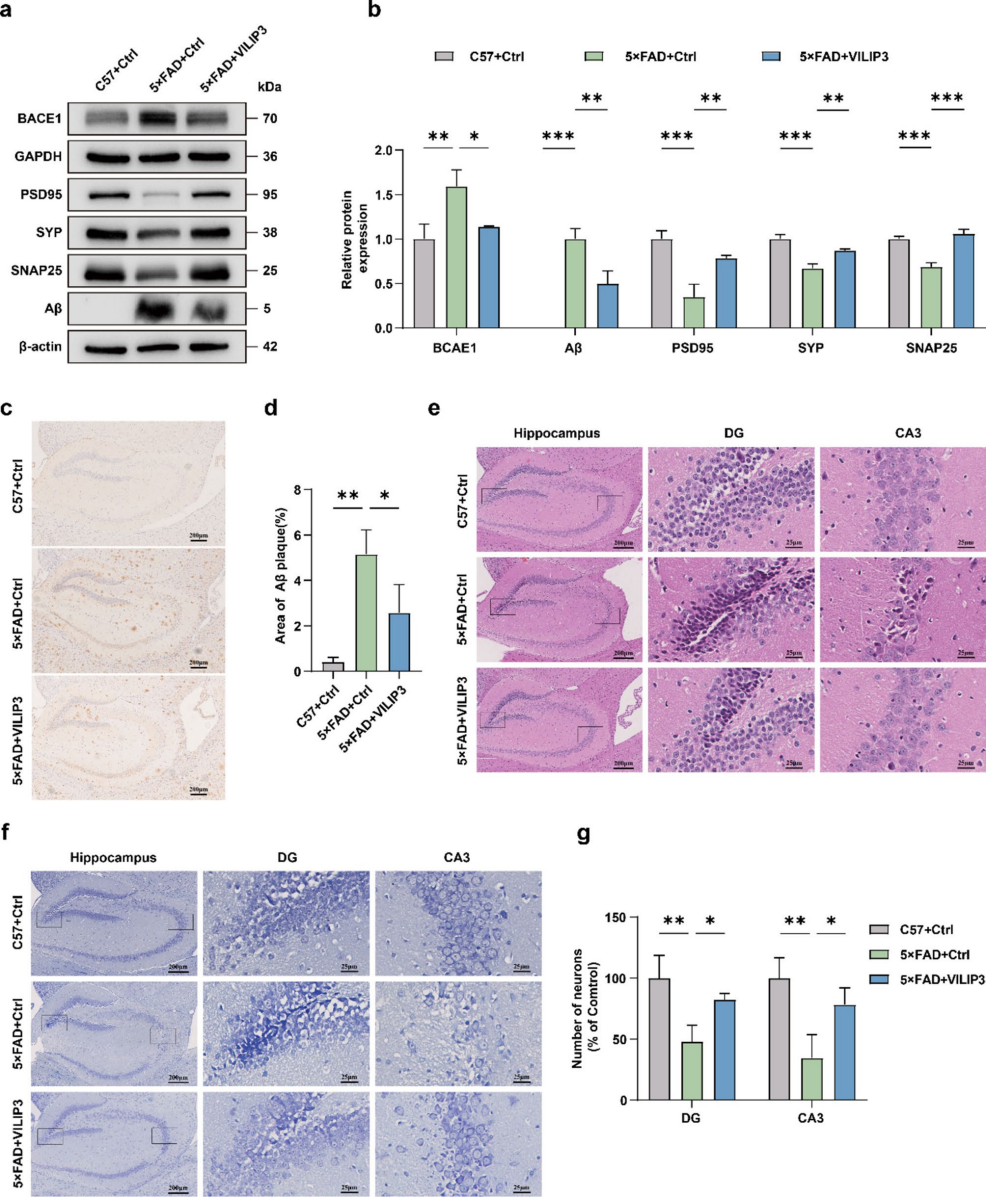
Effects of VILIP3 overexpression on neuropathology in 5×FAD mice.** a**,** b** Relative expression of BACE1, Aβ, PSD95, SYP, and SNAP25 in the hippocampus of mice in the C57 + Ctrl, 5×FAD + Ctrl, and 5×FAD + VILIP3 groups, as determined using WB (*n* = 3). **c**,** d** Representative images and relative expression of Aβ plaques in the hippocampus of mice in the C57 + Ctrl, 5×FAD + Ctrl, and 5×FAD + VILIP3 groups, determined via IHC (Scale bars, 200 μm) (*n* = 3). **e** Representative images of HE staining of the hippocampus of mice in the C57 + Ctrl, 5×FAD + Ctrl, and 5×FAD + VILIP3 groups. The two pictures on the right (Scale bars, 25 μm) are enlargements of the one on the left (Scale bars, 200 μm). **f** Representative images of Nissl staining of the hippocampus of mice in the C57 + Ctrl, 5×FAD + Ctrl, and 5×FAD + VILIP3 groups. The two pictures on the right (Scale bars, 25 μm) are enlargements of the one on the left (Scale bars, 200 μm). **g** Quantification of the number of neurons in the DG and CA3 regions of the mice hippocampus in Nissl staining (*n* = 3). Data are presented as means ± SD. * *p* < 0.05, ** *p* < 0.01, and *** *p* < 0.001

Having concluded that overexpression of VILIP3 rescued cognitive deficits in 5×FAD mice, we further examined its effects on mice neuropathology. Aβ has long been considered a driver of the pathological process in AD, and has an important regulatory role in cognitive loops (Xiao et al. 2023; Zhang et al. 2023). Aβ production requires hydrolysis by β-secretase and γ-secretase, and BACE1 and PS1 are part of β- and γ-secretase, respectively (Long et al. 2021). WB results from mice hippocampus showed that Aβ, BACE1 and PS1 protein expression was significantly higher in the 5×FAD-Ctrl group than in the C57-Ctrl group, whereas VILIP3 overexpression reduced the expression of Aβ and BACE1 in the brains of 5×FAD mice, but did not affect the expression of PS1 (Fig. 3a, b and Fig. S2a, b). To further investigate the effect of VILIP3 on pathological Aβ deposition, we observed the deposition of Aβ plaques using 6E10 antibody staining. The results showed that a large number of brownish-yellow Aβ plaques were deposited in the hippocampus of mice in the 5×FAD-Ctrl group, and the deposition area was significantly larger than that of the C57-Ctrl group, whereas after VILIP3 overexpression, the area of brownish-yellow Aβ plaques in the hippocampus of 5×FAD mice was significantly reduced, and this result was consistent with the data from WB (Fig. 3c, d).

Hippocampal neuron loss is an important pathological manifestation of AD and is associated with cognitive decline (Song et al. 2024). HE staining and Nissl staining showed the overall status of hippocampal neurons in mice. The results demonstrated that the neurons in the C57-Ctrl group exhibited regular morphology, tight arrangement, clear nucleoli, uniform chromatin distribution, and abundant Nissl bodies. In contrast, the neurons in the 5×FAD-Ctrl group displayed disorganization, characterized by irregular cellular morphology. Some of the cells exhibited features indicative of nuclear consolidation, disappearance of nucleoli, and deepening of cytoplasmic staining. Furthermore, a significant decrease in the number of Nissl bodies in the 5×FAD-Ctrl group was observed, suggesting substantial neuronal damage. Following the expression of VILIP3, a significant improvement in neuronal damage was observed in 5×FAD mice, particularly in the DG and CA3 regions (Fig. 3e-g). These findings suggest that the overexpression of VILIP3 may have a positive effect on the neuronal damage experienced by 5×FAD mice. In addition, WB showed that the expression of synapse-associated proteins (PSD95, SYP, and SNAP25) was significantly lower in the 5×FAD-Ctrl group than in the C57-Ctrl group, indicating impaired synaptic function, which was restored after overexpression of VILIP3 in the 5×FAD-VILIP3 group (Fig. 3a, b). Our findings indicate that overexpression of VILIP3 reduced Aβ deposition, attenuated neuronal damage, and improved synaptic function in 5×FAD mice.

### VILIP3 reduces apoptosis and oxidative stress in 5×FAD mice

**Fig. 4:**
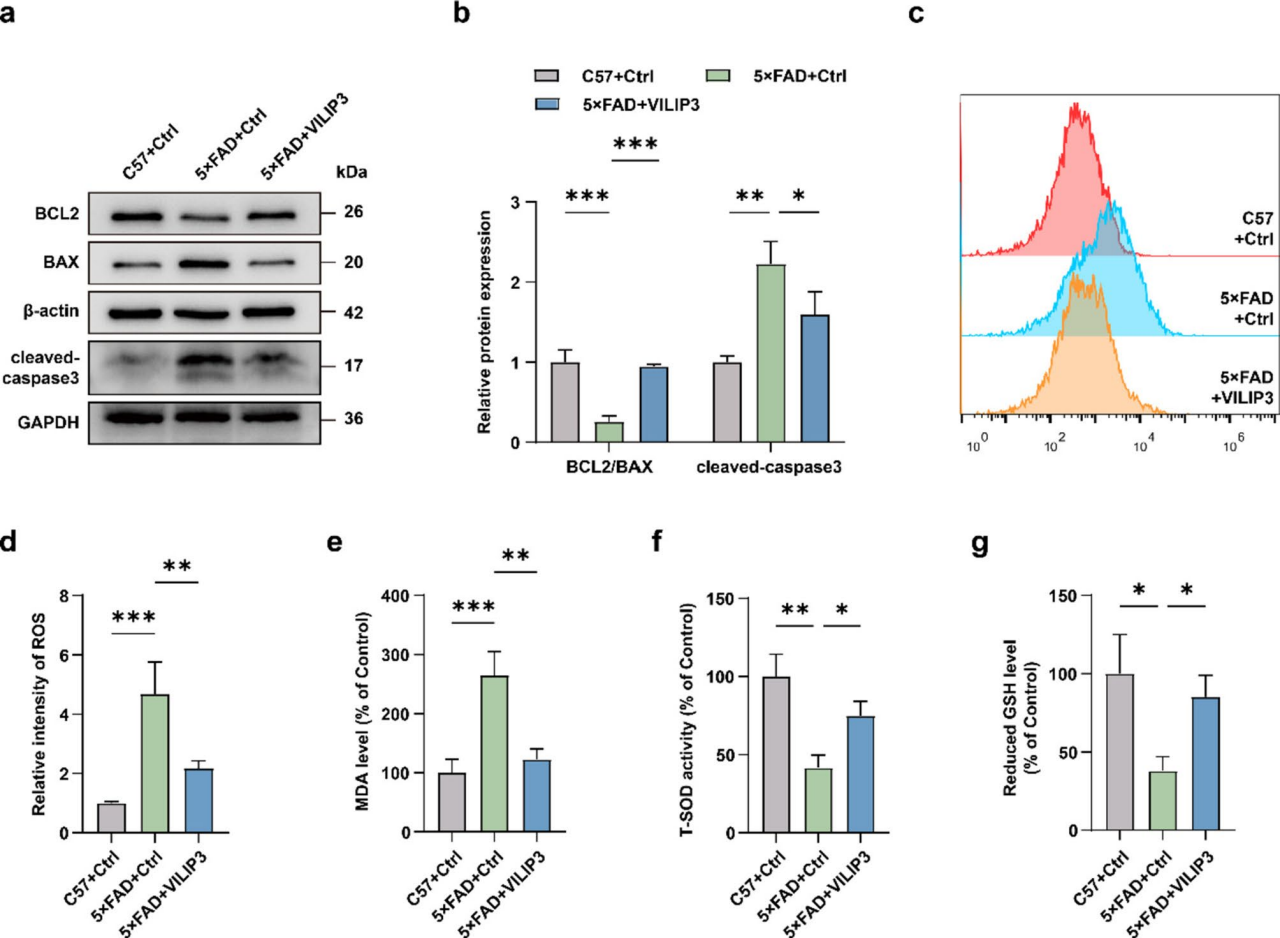
Effects of VILIP3 overexpression on apoptosis and oxidative stress in 5×FAD mice. **a**,** b** Relative expression of BCL2, BAX, and cleaved-caspase3 in the hippocampus of mice in the C57 + Ctrl, 5×FAD + Ctrl, and 5×FAD + VILIP3 groups, as determined using WB (*n* = 3). **c**,** d** Relative intensity of ROS in the hippocampus of mice in the C57 + Ctrl, 5×FAD + Ctrl, and 5×FAD + VILIP3 groups, as determined using flow cytometry (*n* = 3). **e-g** Relative levels of oxidative stress indicators (MDA, T-SOD, and reduced GSH) in the hippocampus of mice in the C57 + Ctrl, 5×FAD + Ctrl, and 5×FAD + VILIP3 groups (*n* = 3). Data are presented as means ± SD. *p < 0.05, **p < 0.01, and ***p < 0.001

Based on our findings regarding the effect of VILIP3 on neuronal loss, we analyzed the expression of apoptosis-related proteins in mouse hippocampal lysates. WB results showed that the ratio of the expression of the anti-apoptotic protein BCL2 to the pro-apoptotic protein BAX in the 5×FAD-Ctrl group was significantly lower than that in the C57-Ctrl group, whereas the expression of cleaved-caspase3 was significantly higher than that in the C57-Ctrl group. However, compared with the 5×FAD-Ctrl group, overexpression of VILIP3 in 5×FAD mice upregulated the ratio of BCL2/BAX expression and downregulated cleaved-caspase3 expression (Fig. 4a, b). These results demonstrated that overexpression of VILIP3 reduced apoptosis in 5×FAD mice. To verify the effect of VILIP3 on oxidative stress, we measured the levels of ROS, T-SOD, reduced GSH, and the lipid peroxidative product (MDA) in the mice hippocampus. The levels of ROS and MDA were significantly higher in the 5×FAD-Ctrl group than in the C57-Ctrl group (Fig. 4c-e), whereas the activity of T-SOD and the levels of reduced GSH were significantly lower than those in the C57-Ctrl group (Fig. 4f, g). After overexpression of VILIP3 in 5×FAD mice, the levels of ROS and MDA were decreased, whereas the activity of T-SOD and the levels of reduced GSH were increased (Fig. 4c-g). These results illustrate that overexpression of VILIP3 ameliorates apoptosis and oxidative stress in 5×FAD mice.

### VILIP3 reduces apoptosis and oxidative stress in SH-SY5Y cells induced by Aβ_1−42_ in vitro

**Fig. 5:**
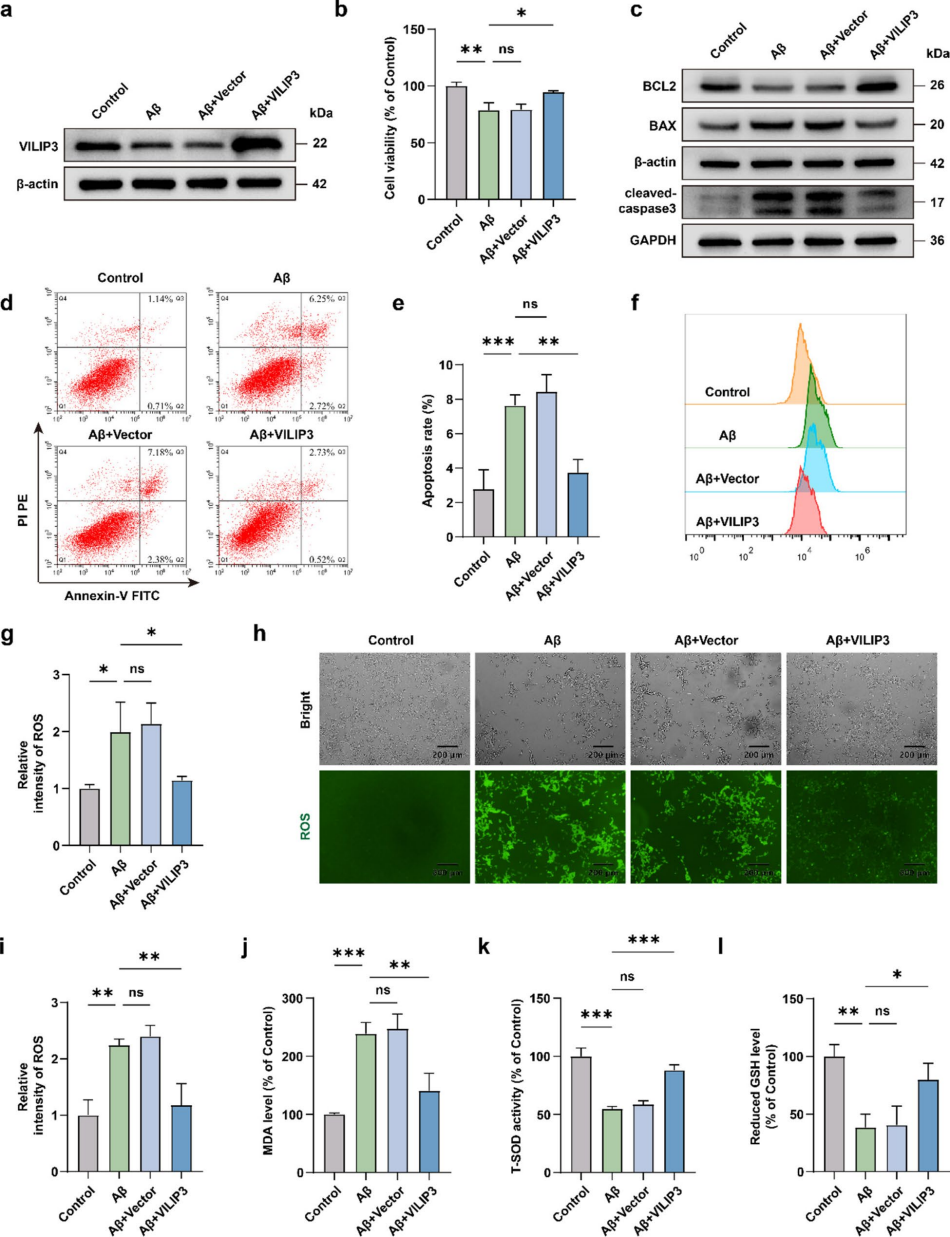
Effects of VILIP3 overexpression on apoptosis and oxidative stress in SH-SY5Y cells.** a** Relative expression of VILIP3 in SH-SY5Y cells in the Control, Aβ, Aβ + Vector, and Aβ + VILIP3 groups, as determined via WB (*n* = 3). **b** Relative cell viability of SH-SY5Y cells in the Control, Aβ, Aβ + Vector and Aβ + VILIP3 groups (*n* = 3). **c** Relative expression of BCL2, BAX, and cleaved-caspase3 in SH-SY5Y cells in the Control, Aβ, Aβ + Vector and Aβ + VILIP3 groups, as determined via WB (*n* = 3). **d**,** e** Apoptosis rate of SH-SY5Y cells in the Control, Aβ, Aβ + Vector, and Aβ + VILIP3 groups, as determined using flow cytometry (*n* = 3). **f**,** g** Relative intensity of ROS in SH-SY5Y cells in the Control, Aβ, Aβ + Vector, and Aβ + VILIP3 groups, as determined using flow cytometry (*n* = 3). **h**,** i** Relative intensity of ROS in SH-SY5Y cells in the Control, Aβ, Aβ + Vector, and Aβ + VILIP3 groups, as determined using IF (Scale bars, 200 μm) (*n* = 3). **j-l** Relative levels of indicators of oxidative stress (MDA, T-SOD, and reduced GSH) in SH-SY5Y cells in the Control, Aβ, Aβ + Vector and Aβ + VILIP3 groups (*n* = 3). Data are presented as means ± SD. *p < 0.05, **p < 0.01 and ***p < 0.001; ns, no significance

We observed a decrease in VILIP3 expression in a cell model of AD in vitro. Subsequently, we constructed a VILIP3 overexpression plasmid and transfected it into SH-SY5Y cells. RT-qPCR and WB results confirmed that VILIP3 was successfully regulated at the mRNA and protein levels (Fig. 5a and Fig. S3a, b). Subsequently, we assessed cell viability using the CCK8 kit. Viability was reduced in Aβ_1−42_-treated SH-SY5Y cells compared to that in the control group, whereas overexpression of VILIP3 rescued cell viability (Fig. 5b). The results of WB showed that the ratio of the expression of the anti-apoptotic protein BCL2 to the pro-apoptotic protein BAX was reduced, and the expression of cleaved-caspase3 was increased in the Aβ-treated group compared to that in the control group. Overexpression of VILIP3 reversed these changes (Fig. 5c and Fig. S3c). Flow cytometry visually demonstrated the apoptosis rate in the different treatment groups. The results showed that the apoptosis rate of Aβ_1−42_-treated SH-SY5Y cells was higher than that of the control group, whereas the apoptosis rate was reduced after overexpression of VILIP3 (Fig. 5d, e). Intracellular ROS and MDA levels were significantly higher in the Aβ group than in the control group, whereas the levels of ROS and MDA were significantly lower in the Aβ + VILIP3 group than in the Aβ group (Fig. 5f-j). In contrast, T-SOD viability and reduced GSH levels of cells in the Aβ-treated group were significantly lower than those in the control group, and overexpression of VILIP3 in the Aβ group rescued their reduction (Fig. 5k, l). These results suggest that VILIP3 can reduce apoptosis and oxidative stress in an in vitro AD model, which is consistent with in vivo results.

### VILIP3 provides antioxidant protection by activating the Nrf2 signaling pathway

**Fig. 6:**
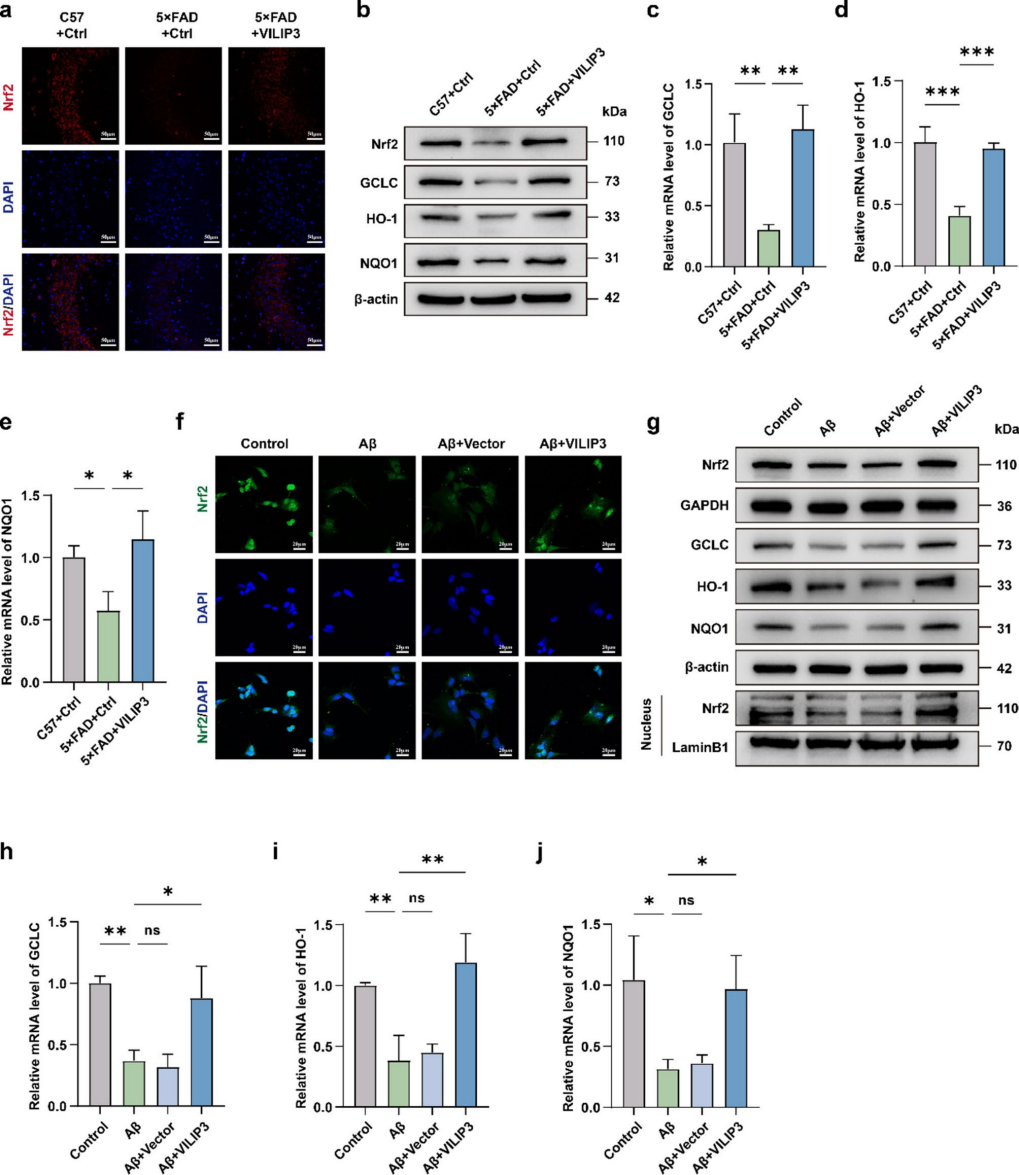
Effects of VILIP3 overexpression on Nrf2 signaling pathway in vivo and in vitro.** a** Relative intensity of Nrf2 in the hippocampus of mice in the C57 + Ctrl, 5×FAD + Ctrl, and 5×FAD + VILIP3 groups, as determined using IF (Scale bars, 50 μm). **b** Relative expression of Nrf2, GCLC, HO-1, and NQO1 in the hippocampus of mice in the C57 + Ctrl, 5×FAD + Ctrl, and 5×FAD + VILIP3 groups, as determined using WB. **c-e** Relative mRNA levels of GCLC, HO-1, and NQO1 in the hippocampus of mice in the C57 + Ctrl, 5×FAD + Ctrl, and 5×FAD + VILIP3 groups, as determined using RT-qPCR (*n* = 3). **f** Relative intensity of Nrf2 in SH-SY5Y cells in the Control, Aβ, Aβ + Vector, and Aβ + VILIP3 groups, as determined via IF (Scale bars, 20 μm). **g** Relative expression of GCLC, HO-1, NQO1, total Nrf2, and nuclear Nrf2 in SH-SY5Y cells in the Control, Aβ, Aβ + Vector, and Aβ + VILIP3 groups, as determined via WB. **h-j** Relative mRNA levels of GCLC, HO-1, and NQO1 in SH-SY5Y cells in the Control, Aβ, Aβ + Vector, and Aβ + VILIP3 groups, as determined via RT-qPCR (*n* = 3). Data are presented as means ± SD. *p < 0.05, **p < 0.01 and ***p < 0.001; ns, no significance

Nrf2 and related pathways are vital in oxidative stress, and the treatment of AD by activating the Nrf2 pathway holds great promise (Osama et al. 2020). Based on the role of VILIP3 in regulating oxidative stress, we investigated whether VILIP3 exerts its antioxidant effects by influencing the Nrf2 signaling pathway. We first examined the expression levels of Nrf2 in mice hippocampal tissues by IF and WB. The results showed that Nrf2 levels were significantly reduced in the 5×FAD-Ctrl group compared to the C57-Ctrl group, while overexpression of VILIP3 in 5×FAD mice increased Nrf2 expression levels (Fig. 6a, b and Fig. S4a, b). We then examined the expression levels of antioxidant genes downstream of Nrf2 in mice hippocampal tissue to determine whether the increased Nrf2 expression implied reactivation of the Nrf2 pathway. The results of RT-qPCR and WB showed that the mRNA and protein levels of GCLC, HO-1 and NQO1 were significantly reduced in the hippocampus of mice in the 5×FAD-Ctrl group compared with those in the C57-Ctrl group; whereas the mRNA and protein levels of these antioxidant genes were significantly upregulated after overexpression of VILIP3 in 5×FAD mice, indicating successful activation of the Nrf2 pathway (Fig. 6b-e and Fig. S4b). We then investigated the changes in the Nrf2 pathway in the in vitro model of AD and found that Nrf2 expression and its intranuclear levels were significantly reduced in cells from the Aβ_1−42_-treated group compared to that in the control group, and overexpression of VILIP3 partially restored Nrf2 activity (Fig. 6f, g and Fig. S4c-e). We also found that treatment of SH-SY5Y cells with Aβ_1−42_ resulted in a significant reduction in the mRNA and protein levels of NQO1, HO-1, and GCLC, while overexpression of VILIP3 partially restored their expression (Fig. 6h-j and Fig. S4e). This suggests that activation of the Nrf2 pathway may be positively correlated with VILIP3 expression in AD models.

### Inhibition of Nrf2 reverses the neuroprotective effect of overexpression of VILIP3

**Fig. 7:**
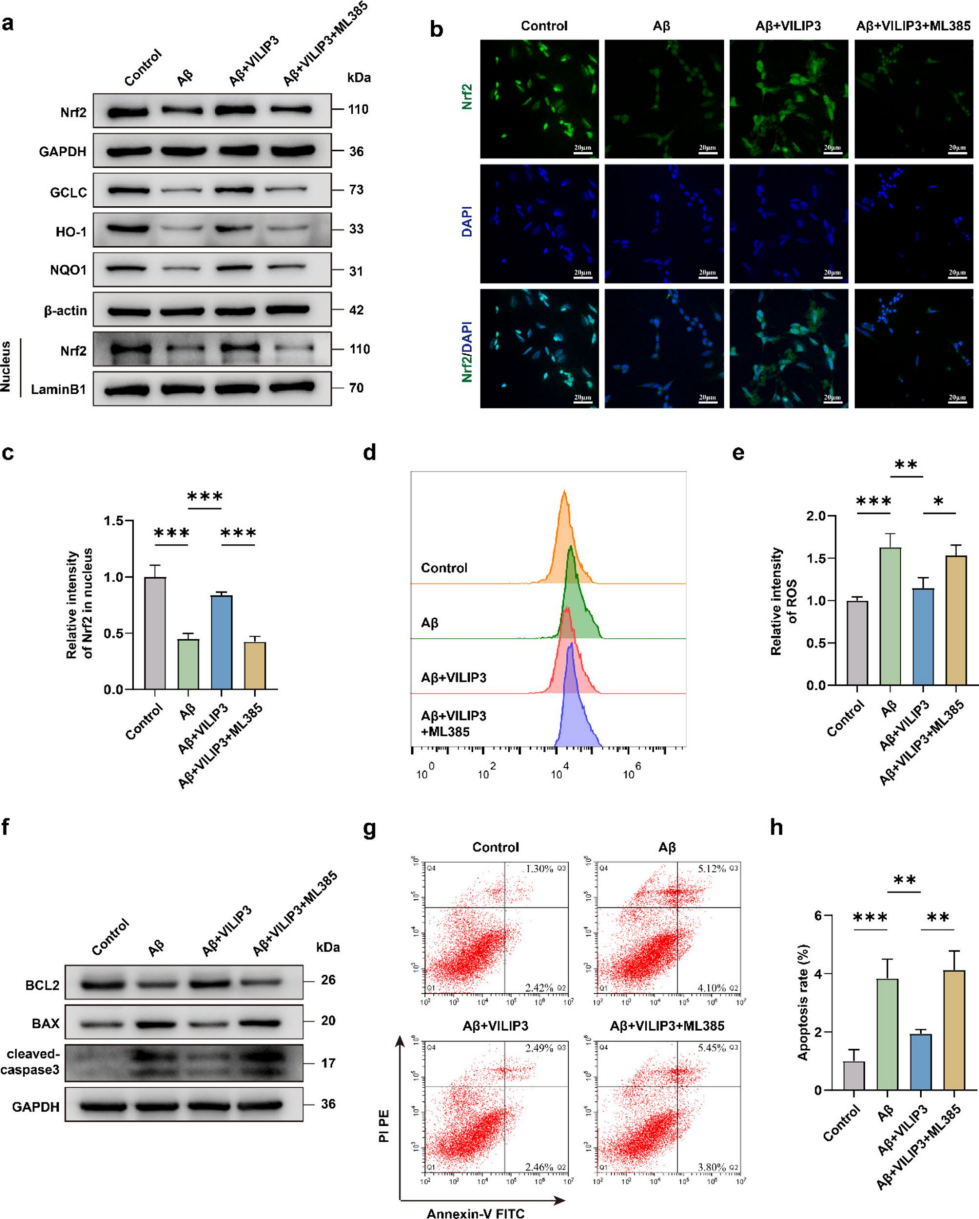
Effects of the Nrf2 inhibitor on the function of VILIP3 in SH-SY5Y cells.** a** Relative expression of total Nrf2, nuclear Nrf2, GCLC, HO-1, and NQO1 in SH-SY5Y cells in the Control, Aβ, Aβ + VILIP3, and Aβ + VILIP3 + ML385 groups, as determined via WB. **b**,** c** Relative intensity of Nrf2 in SH-SY5Y cells in the Control, Aβ, Aβ + VILIP3, and Aβ + VILIP3 + ML385 groups, as determined via IF (Scale bars, 20 μm) (*n* = 3). **d**,** e** Relative intensity of ROS in SH-SY5Y cells in the Control, Aβ, Aβ + VILIP3, and Aβ + VILIP3 + ML385 groups as determined using flow cytometry (*n* = 3). **f** Relative expression of BCL2, BAX, and cleaved-caspase3 in SH-SY5Y cells in the Control, Aβ, Aβ + VILIP3, and Aβ + VILIP3 + ML385 groups, as determined via WB. **g**,** h** Apoptosis rate of SH-SY5Y cells in the Control, Aβ, Aβ + VILIP3, and Aβ + VILIP3 + ML385 groups, as determined using flow cytometry (*n* = 3). Data are presented as means ± SD. *p < 0.05, **p < 0.01 and ***p < 0.001

To further confirm that VILIP3 exerts neuroprotective effects through Nrf2, an Nrf2 inhibitor, ML385, was used in Aβ_1−42_-treated SH-SY5Y cells overexpressing VILIP3. The addition of ML385 decreased the expression level of Nrf2 compared to the Aβ + VILIP3 group, suggesting that ML385 successfully inhibited the Nrf2 signaling pathway (Fig. a-c and Fig. S5a). Meanwhile, the addition of ML385 decreased the expression of antioxidant genes downstream of Nrf2 and increased the level of intracellular ROS compared to that in the Aβ + VILIP3 group (Fig. 7a, d,e and Fig. S5b). This suggests that the addition of ML385 counteracted the role of VILIP3 in the antioxidant response. Furthermore, the results of WB and flow cytometry showed that the addition of ML385 decreased the BCL2/BAX expression ratio, increased the expression of cleaved-caspase3, and upregulated the apoptosis rate, masking the protective effect of VILIP3 against apoptosis in Aβ_1−42_-treated and VILIP3-overexpressing SH-SY5Y cells (Fig. 7f-h and Fig. S5c). These results further validate the relevance of VILIP3 to the Nrf2 signaling pathway in AD models.

## Discussion

Based on the prevalence and severity of AD, achieving an in-depth understanding of its pathogenesis and identifying effective therapeutic targets have been major research challenges in recent years. (Zhang et al. 2023). VILIP3, a member of the VSNL subfamily of NCS proteins, is widely expressed in neurons but not glias in the human brain (Bernstein et al. 1999; Spilker 2003; Spilker et al. 2002). Another member of the VSNL subfamily, VILIP1, is elevated in the cerebrospinal fluid of patients with AD and has been suggested as a potential biomarker for the early diagnosis of AD (Halbgebauer et al. 2022). However, the role of VILIP3 in AD remains underexplored. In this study, we found that VILIP3 expression is reduced in animal and cellular models of AD. Overexpression of VILIP3 ameliorated cognitive deficits and neuropathological impairments in 5×FAD mice, attenuating neuronal apoptosis and oxidative stress levels by activating the Nrf2 signaling pathway to exert neuroprotective effects. These findings suggest that VILIP3 may serve as a potential therapeutic target for AD.

Several studies have shown that 5×FAD mice develop neuropathological changes at 2 months of age and exhibit impaired spatial memory at 4–6 months of age (Forner et al. 2021; Pádua et al. 2024). VILIP3 has been partially detected extracellularly in the brains of patients with AD in association with the neuritic plaques (Braunewell et al. 2001), a subset of Aβ plaques containing dystrophic neurites, a typical pathological feature of AD (Tsering et al. 2023). Therefore, we selected 3- and 6-month-old mice for our study, focusing on Aβ pathology. In this study, we found that VILIP3 expression was downregulated in the hippocampus of 5×FAD mice and in Aβ_1−42_-interfered SH-SY5Y cells, which is consistent with previous reports of reduced VILIP3 expression in neocortical areas of AD patients in clinical studies (Braunewell et al. 2001), suggesting that abnormal VILIP3 expression may be a general feature of AD pathology. In addition, no differences in VILIP3 expression were observed in the hippocampus of 3-month-old mice in the present study, whereas in in vitro experiments VILIP3 showed a dose-dependent decrease in response to Aβ_1−42_, suggesting that VILIP3 is not yet affected in the early stages of AD but may be involved in disease progression.

To further understand the role of VILIP3 in AD pathology, this study then explored the effects of VILIP3 on cognitive dysfunction and neuropathology in AD. We found that VILIP3 alleviated cognitive deficits in AD mice, highlighting its clinical relevance as progressive cognitive decline is a core AD symptom and major contributor to daily functional decline (Scheltens et al. 2021). From the perspective of pathological mechanisms, we found that VILIP3 significantly reduced Aβ deposition and neuronal loss, and increased the expression of synaptic functional proteins to restore synaptic function. Aβ deposition arises from APP cleavage by α-, β- (BACE1), and γ-secretases (Zhang et al. 2011). BACE1, the rate-limiting enzyme in Aβ production, is a key therapeutic target due to its dysregulation in AD (Bazzari et al. 2022). Notably, VILIP3 overexpression selectively reduced hippocampal BACE1 activity in 5×FAD mice without affecting γ-secretase (PS1), suggesting specific inhibition of the amyloidogenic APP pathway via BACE1 modulation. The results of the present study showed that SNAP25, SYP and PSD95, which are critical for neurotransmitter exocytosis/endocytosis (Ali et al. 2023), were reduced in the brains of 5×FAD mice, consistent with previous findings (Wang et al. 2024, 2025), but VILIP3 overexpression restored their expression, suggesting that it may improve cognitive function by maintaining synaptic structural and functional integrity. As a member of the NCS protein family, the widespread distribution of VILIP3 in neuronal soma, dendrites, and axons suggests its potential role in regulating synaptic function through modulation of calcium signaling pathway (Bernstein et al. 1999; Spilker et al. 2002). Some NCS proteins (e.g. calsenilin, VILIP2, hippocalcin and NCS-1) have been shown to regulate synaptic plasticity, and VILIP3 may have a similar functional mechanism with them (Braunewell 2012; Braunewell et al. 2009), which is worth further investigation.

VILIP3 may influence the redox reactions in AD, but direct evidence is currently lacking (Oikawa et al. 2016). In this study, we found that VILIP3 was able to attenuate oxidative stress and apoptosis in AD models, and this dual protective mechanism has important physiological significance in AD. There is a pathological vicious circle between oxidative stress and Aβ deposition (Bai et al. 2022; Liu et al. 2023). Elevated levels of oxidative stress can trigger lipid peroxidation, leading to the production of large amounts of MDA and further cellular dysfunction (Lee et al. 2024). In this study, overexpression of VILIP3 reduced the accumulation of ROS and MDA in AD models, which reflected the attenuation of oxidative stress levels, suggesting its blocking effect on the oxidative damage cascade. In this study, we also found that overexpression of VILIP3 enhanced T-SOD activity and increased the level of reduced GSH in AD models. T-SOD and reduced GSH are important endogenous antioxidant components in vivo, suggesting that VILIP3 can maintain redox homeostasis by enhancing the antioxidant defense system. However, a study in tumor tissue and pancreatitis models found that VILIP3 could promote MDA accumulation by enhancing ferroptosis-related lipid peroxidation (Chen et al. 2023). The emergence of such differences may be due to differences in disease models and molecular interaction networks, suggesting a tissue microenvironment-dependent function of VILIP3. Apoptosis is an important cause of neuronal loss, and studies have reported that Aβ deposition triggers the apoptotic pathway, leading to neuronal death (Kumari et al. 2023). The results of our study showed that overexpression of VILIP3 reversed the changes in the expression of BCL2, BAX and cleaved-caspase3 in AD and reduced the proportion of apoptotic cells. Those results demonstrate the anti-apoptotic effect of VILIP3 in AD and suggest that it may protect neurons by regulating the mitochondrial apoptotic pathway. Previous studies have shown that VILIP3 promotes BCL2 expression in hepatocellular carcinoma cells (Tan et al. 2023), which is consistent with our findings. The effects of VILIP3 on oxidative stress and neuronal apoptosis may be a potential mechanism for its amelioration of neuropathological damage.

Based on the close association of VILIP3 with oxidative stress and apoptosis in AD, we further explored the downstream signaling pathway in which VILIP3 functions. We found that VILIP3 was able to activate the Nrf2 signaling pathway in AD models and effectively reduced the levels of oxidative stress and apoptosis by upregulating the expression of antioxidant response element (ARE)-related genes (NQO1, HO-1, GCLC). This finding provides a new molecular basis for elucidating the neuroprotective mechanism of VILIP3. Nrf2 is a cytoprotective transcription factor that primarily safeguards cellular redox homeostasis by regulating the ARE pathway (He et al. 2020). Numerous studies have shown that Nrf2 is impaired in AD, that lack of Nrf2 exacerbates cognitive deficits, and that activation of Nrf2 reduces Aβ expression and ameliorates oxidative stress and neuroinflammation (Branca et al. 2017; Uruno et al. 2020; Yu et al. 2019). Nrf2 exerts transcriptional regulation primarily in the nucleus, and its subcellular location and molecular activity are subject to complex regulation. Kelch-like ECH-associated protein 1 (Keap1) is a major regulator of Nrf2. Additionally, changes in the levels or activities of GSK3β, Bach1, p53, and Hrd1 may also affect Nrf2 activity (Silva-Palacios et al. 2018). In this study, VILIP3 was found to be a novel activator of Nrf2, but the mechanism by which VILIP3 activates Nrf2 needs further investigation, and crosstalk between VILIP3 and GSK3β and Nrf2 may be one of the relevant mechanisms, but further evidence is needed. In addition, there is a vicious cycle between oxidative stress and apoptosis. In the progression of AD, excessive ROS can decrease the expression of BCL2 and increase the expression of BAX, thereby affecting the permeability of the mitochondrial membrane and triggering apoptosis. ROS can also activate the caspase family and promote the apoptotic process (Shi et al. 2024; Singh et al. 2024). Therefore, by targeting both oxidative stress and apoptosis through the Nrf2 pathway, VILIP3 may break this vicious cycle and thereby delaying AD progression.

Although our study explored a novel mechanism for the involvement of aberrant VILIP3 expression in AD pathogenesis, some limitations remain. We did not use clinical data for validation, which may affect the generalizability of our findings. The functional study of VILIP3 is relatively simple, and its function needs to be further validated by multidimensional experiments in the future. In addition, we found that VILIP3 may exert neuroprotective effects by modulating the Nrf2 pathway, but its specific molecular mechanism has not been elucidated, and further studies are needed to confirm the molecular properties of VILIP3 and its biological role in AD.

## Conclusions

Our study demonstrated that VILIP3, a differentially expressed NCS protein in AD, ameliorates cognitive deficits and neuropathological impairments during AD, potentially by modulating the Nrf2 signaling pathway to attenuate oxidative stress and reduce neuronal apoptosis. This suggests that VILIP3 may be a potential target for the treatment of AD, and there is a need for more comprehensive studies of the role and mechanism of VILIP3 in AD in the future, as well as the development of specific VILIP3 activators to better support clinical treatment.

## Supplementary Information

Supplementary Material 1.

## Data Availability

No datasets were generated or analysed during the current study.

## Abbreviations

AAV: Adeno-associated virus
AD: Alzheimer’s disease
Aβ: Amyloid-β protein
ARE: Antioxidant response element
BACE1: Beta-site amyloid precursor protein cleaving enzyme 1
BAX: Bcl2 associated X protein
BCA: Bicinchoninic acid
BCL2: B-cell lymphoma-2
CCK8: Cell-Counting-Kit-8
DAPI: 4’,6-diamidino-2-phenylindole
DAB: Diaminobenzidine
ECL: Enhanced chemiluminescence
HE: Hematoxylin-eosin
HO-1: Heme oxygenase-1
IHC: Immunohistochemistry
IF: Immunofluorescence
GCLC: Glutamate-cysteine ligase catalytic subunit
GSH: Glutathione
GSK3β: Glycogen Synthase Kinase 3β
GSS: Glutathione synthetase
MDA: Malondialdehyde
MWM: Morris water maze
NCS: Neuronal Ca^2+^-sensor
NQO1: NADPH quinone oxidoreductase 1
Nrf2: Nuclear factor E2-related factor 2
PBS: Phosphate-buffered saline
PI: Propidium Iodide
PS1: Presenilin 1
PMSF: Phenylmethanesulfonyl fluoride
PVDF: Polyvinylidene fluoride
PSD95: Postsynaptic density-95
RIPA: Radio-immunoprecipitation assay
ROS: Reactive oxygen species
RT-qPCR: Real-time quantitative polymerase chain reaction
T-SOD: Total superoxide dismutase
VILIP3: Visinin-like protein 3
VSNL: Visinin-like protein
WB: Western blot
SD: Standard deviation
SNAP25: Synaptosome Associated Protein 25
SYP: Synaptophysin
