# Supplementary material for: VILIP3 attenuates neuronal apoptosis and oxidative stress via Nrf2 activation in the pathogenesis of Alzheimer’s disease
Source: Mol Med. 2025 Jun 10;31:227. doi: 10.1186/s10020-025-01280-9 (PMC12150439; doi:10.1186/s10020-025-01280-9)
Supplement: Supplementary file 1 — Supplementary Material 1. [file 10020_2025_1280_MOESM1_ESM.docx]

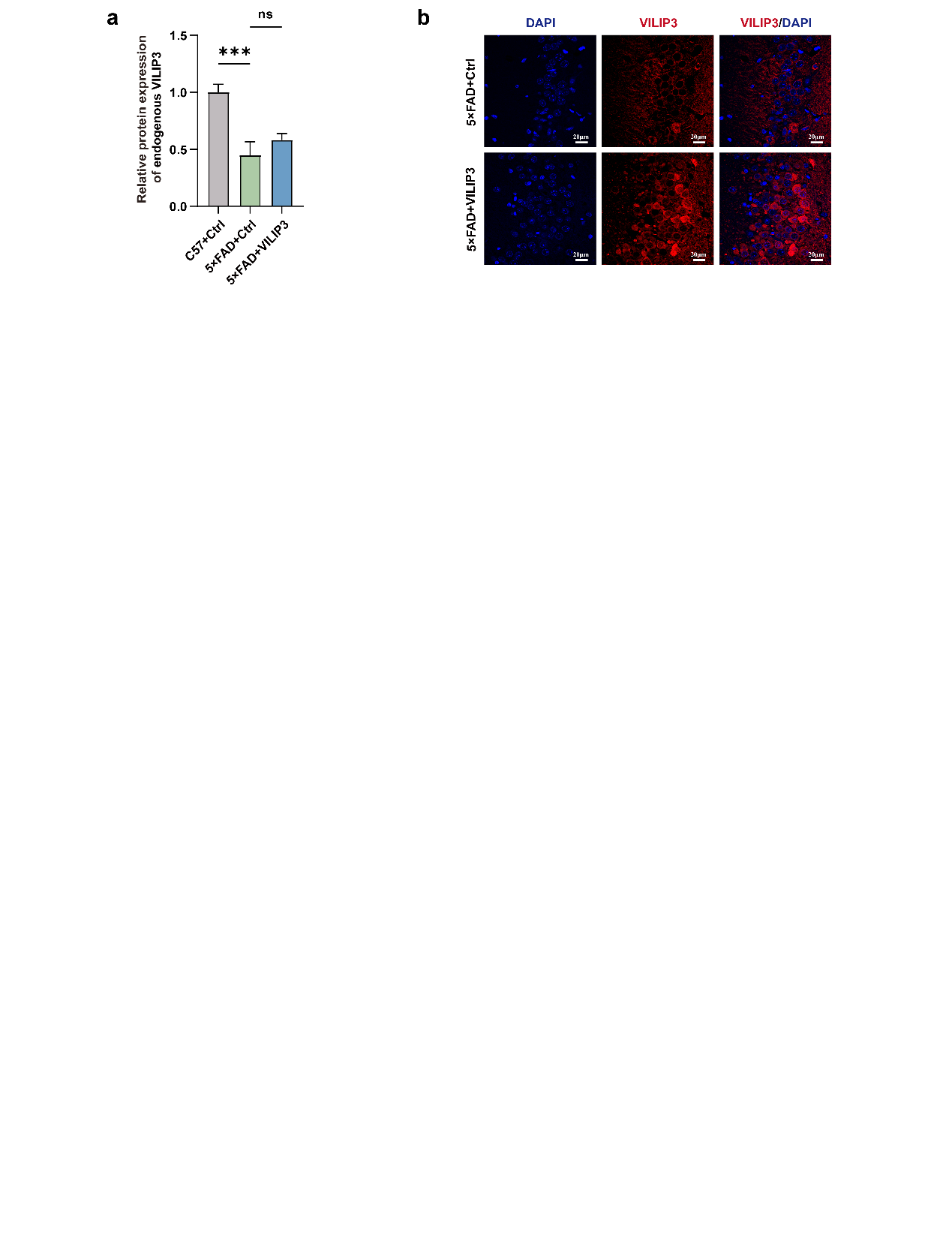


**Fig. S1 Effects of VILIP3 overexpression on cognitive function in 5×FAD mice.** **(a)** Analysis of expression levels of endogenous VILIP3 in the hippocampus of mice in the C57+Ctrl, 5×FAD+Ctrl, and 5×FAD+VILIP3 groups, as determined via WB (n = 3). **(b)** Relative density of VILIP3 in the hippocampus of mice in the C57+Ctrl, 5×FAD+Ctrl, and 5×FAD+VILIP3 groups, as determined using IF (Scale bars, 20 μm). Data are presented as means ± SD. ****p* < 0.001; ns, no significance.


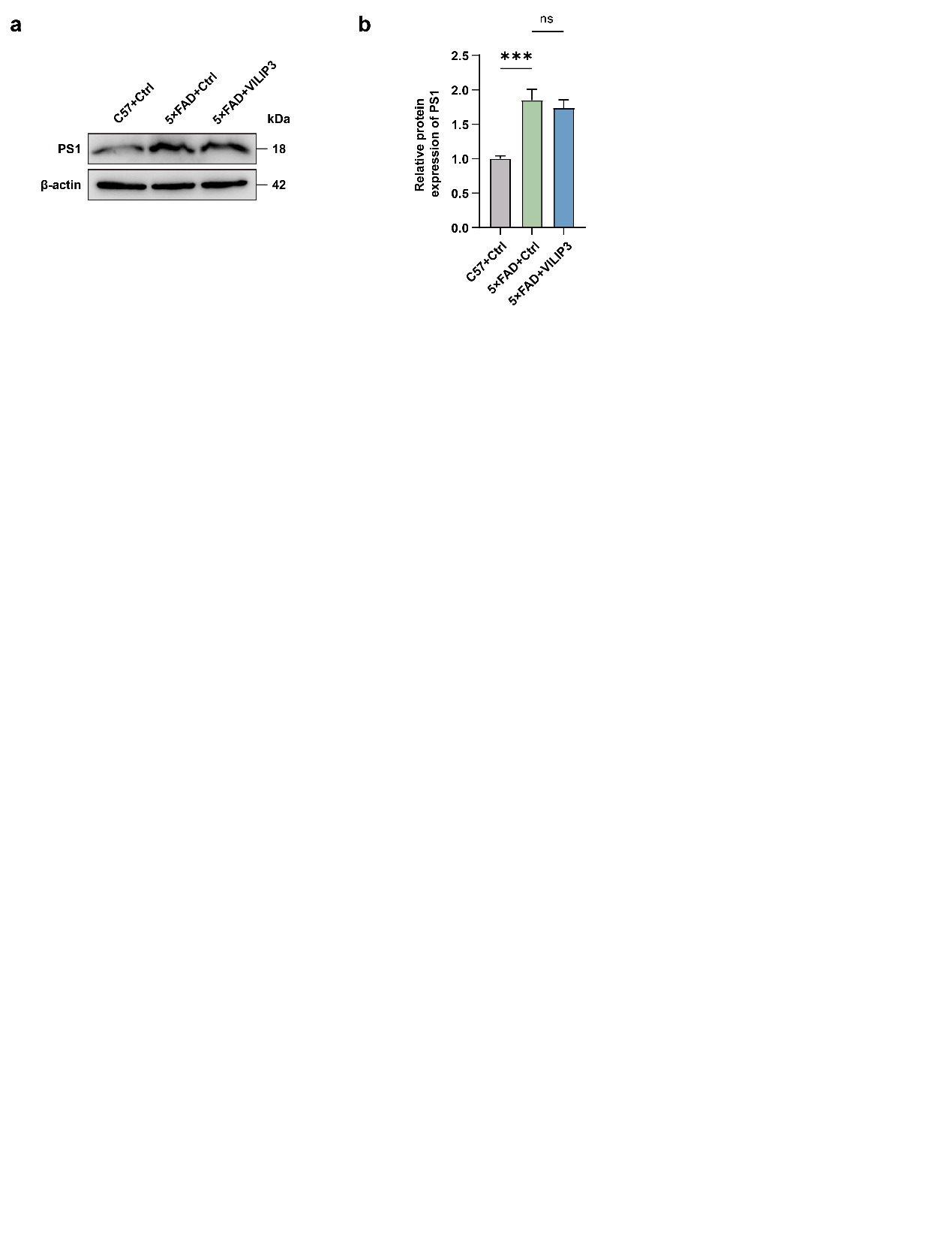


**Fig. S2 Effects of VILIP3 overexpression on neuropathology in 5×FAD mice. (a)(b)** Relative expression of PS1 in the hippocampus of mice in the C57+Ctrl, 5×FAD+Ctrl, and 5×FAD+VILIP3 groups, as determined using WB (n = 3). Data are presented as means ± SD. *** *p* < 0.001; ns, no significance.


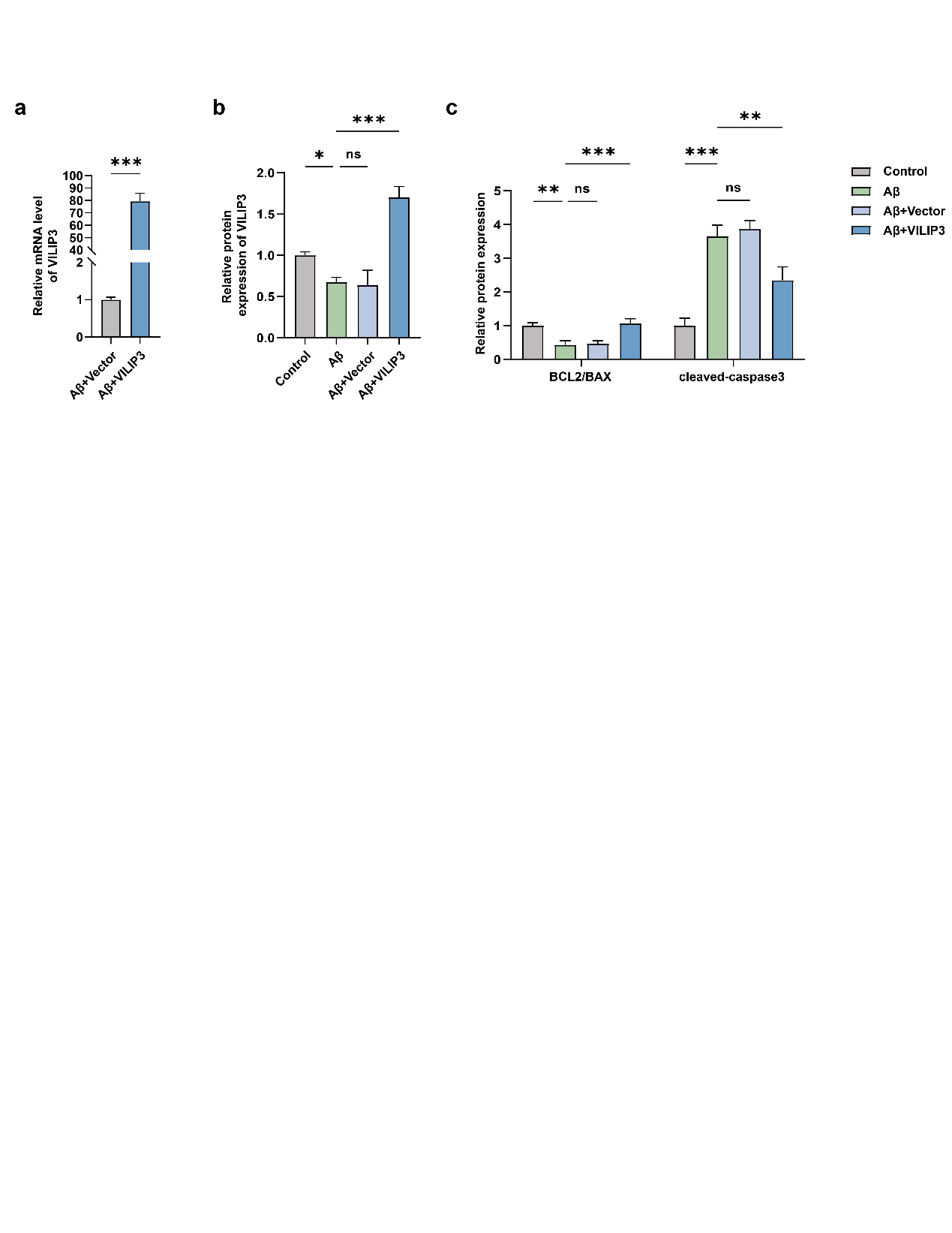


**Fig. S3 Effects of VILIP3 overexpression on apoptosis and oxidative stress in SH-SY5Y cells.** **(a)** Relative VILIP3 mRNA levels in SH-SY5Y cells in the Control, Aβ, Aβ+Vector, and Aβ+VILIP3 groups, as determined via RT-qPCR (n = 3). **(b,c)** Analysis of expression levels of VILIP3, BCL2, BAX, and cleaved-caspase3 in SH-SY5Y cells in the Control, Aβ, Aβ+Vector, and Aβ+VILIP3 groups, as determined through WB (n = 3). Data are presented as means ± SD. **p* < 0.05, ***p* < 0.01 and ****p* < 0.001.


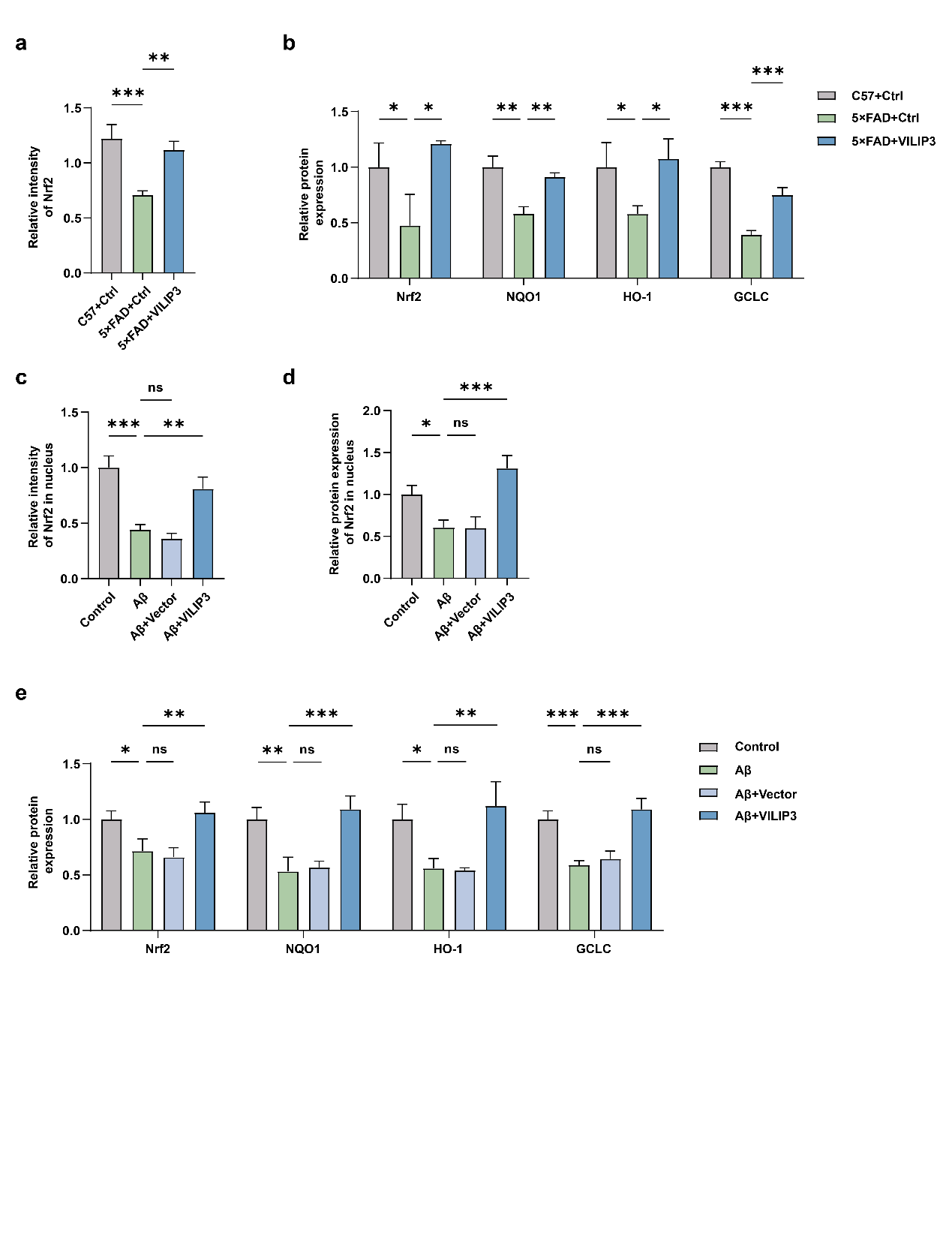


**Fig. S4 Effects of VILIP3 overexpression on the Nrf2 signaling pathway in vivo and in vitro. (a)** Analysis of relative intensity of Nrf2 in the hippocampus of mice in the C57+Ctrl, 5×FAD+Ctrl, and 5×FAD+VILIP3 groups, as determined through IF (n = 3). **(b)** Analysis of expression levels of Nrf2, NQO1, HO-1, and GCLC in the hippocampus of mice in the C57+Ctrl, 5×FAD+Ctrl, and 5×FAD+VILIP3 groups, as determined via WB (n = 3). **(c)** Analysis of relative intensity of nuclear Nrf2 in SH-SY5Y cells in the Control, Aβ, Aβ+Vector, and Aβ+VILIP3 groups, as determined via IF (n = 3). **(d)** Analysis of expression levels of nuclear Nrf2 in SH-SY5Y cells in the Control, Aβ, Aβ+Vector, and Aβ+VILIP3 groups, as determined via WB (n = 3). **(e)** Analysis of expression levels of NQO1, HO-1, GCLC and total Nrf2 in SH-SY5Y cells in the Control, Aβ, Aβ+Vector, and Aβ+VILIP3 groups, as determined via WB (n = 3). Data are presented as means ± SD. **p* < 0.05, ***p* < 0.01 and ****p* < 0.001; ns, no significance.


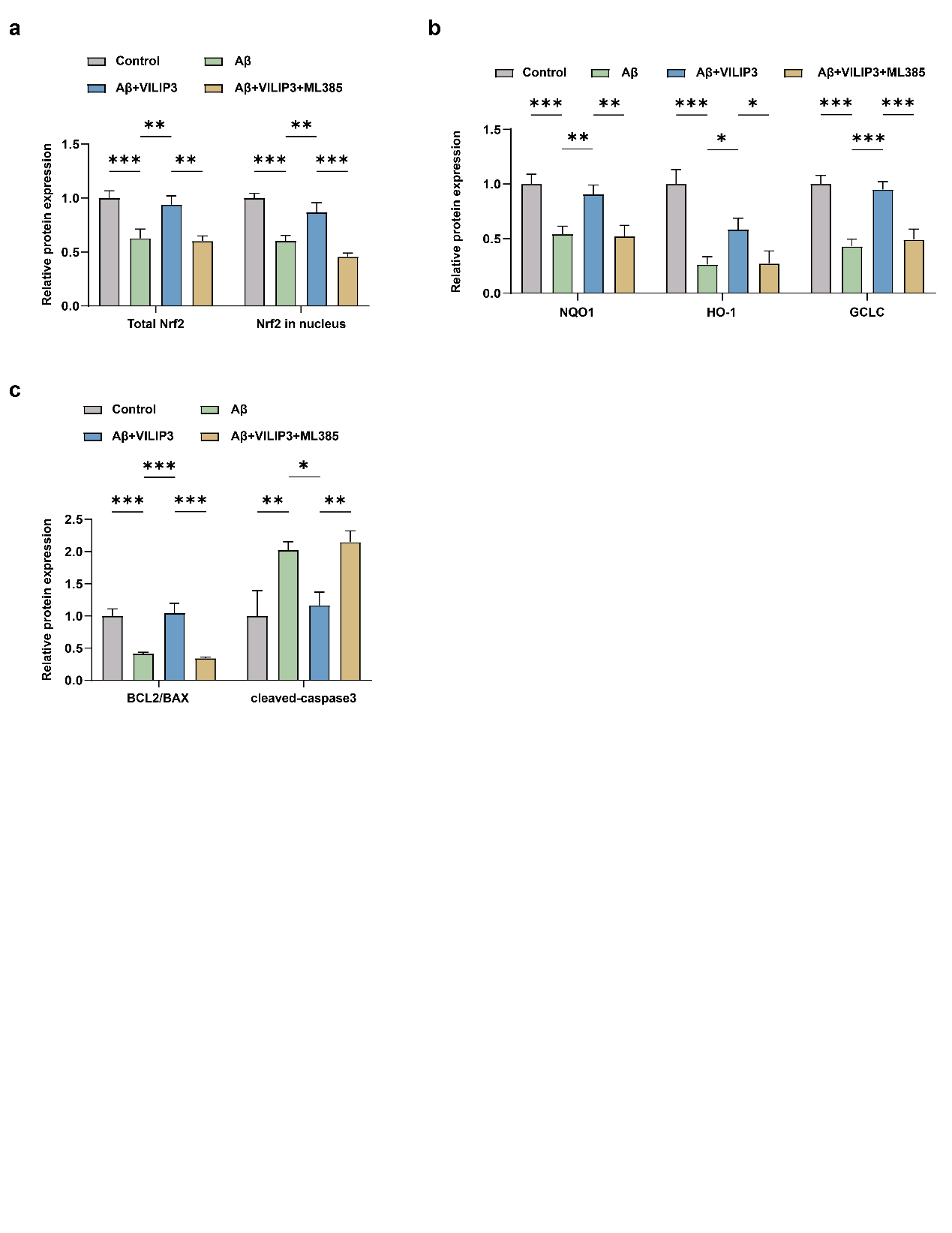


**Fig. S5** **Effects of the Nrf2 inhibitor on the function of VILIP3 in SH-SY5Y cells.** **(a)** Analysis of expression levels of total Nrf2 and nuclear Nrf2 in SH-SY5Y cells in the Control, Aβ, Aβ+VILIP3, and Aβ+VILIP3+ML385 groups, as determined via WB (n = 3). **(b)** Analysis of expression levels of NQO1, HO-1 and GCLC in SH-SY5Y cells in the Control, Aβ, Aβ+VILIP3, and Aβ+VILIP3+ML385 groups, as determined via WB (n = 3). **(c)** Analysis of expression levels of BCL2, BAX, and cleaved-caspase3 in SH-SY5Y cells in the Control, Aβ, Aβ+VILIP3, and Aβ+VILIP3+ML385 groups, as determined through WB (n = 3). Data are presented as means ± SD. **p* < 0.05, ***p* < 0.01 and ****p* < 0.001.

**Table S1 The sequence of** **PcDNA3.1-VILIP3** **and AAV-GFP-VILIP3.**

| **Name** | **Sequence** |
| --- | --- |
| PcDNA3.1-VILIP3 | NCBI Reference Sequence: NM_002149.4  ATGGGCAAACAGAACAGCAAGCTGCGGCCCGAGGTGCTGCAGGACCTGCGGGAGAACACGGAGTTCACCGACCACGAGCTGCAGGAGTGGTACAAGGGCTTCCTCAAGGACTGCCCCACCGGCCACCTGACCGTGGACGAGTTCAAGAAGATCTACGCCAACTTCTTCCCCTACGGCGACGCTTCCAAGTTCGCCGAGCACGTCTTCCGCACCTTCGACACCAACGGCGACGGCACCATCGACTTCCGGGAGTTCATCATTGCGCTGAGCGTGACCTCGCGGGGCAAGCTGGAGCAGAAGCTCAAGTGGGCCTTCAGCATGTACGACCTGGACGGCAACGGCTACATCAGCCGCAGCGAGATGCTGGAGATCGTGCAGGCCATCTACAAGATGGTGTCGTCTGTGATGAAGATGCCGGAGGATGAGTCCACCCCGGAGAAGCGCACAGACAAGATCTTCAGGCAGATGGACACCAACAATGACGGCAAACTGTCCTTGGAAGAATTCATCAGAGGTGCCAAGAGCGACCCCTCCATCGTCCGCCTGCTGCAGTGCGACCCCAGCAGTGCCAGTCAGTTC |
| AAV-GFP-VILIP3 | NCBI Reference Sequence: NM_016677.5  ATGGGCAAGCAGAACAGCAAGCTGCGTCCTGAGGTGCTGCAGGACCTGCGGGAACACACGGAATTCACCGACCATGAGCTTCAGGAGTGGTACAAGGGCTTCCTCAAGGACTGCCCCACTGGCCACCTGACTGTGGACGAGTTCAAGAAGATCTACGCCAACTTCTTCCCCTACGGCGATGCCTCCAAGTTCGCTGAACACGTCTTCCGCACCTTCGACACCAACAGCGATGGCACCATCGACTTCCGGGAGTTCATCATTGCTCTGAGTGTGACCTCTCGGGGCAAGCTGGAACAGAAGCTCAAGTGGGCCTTTAGCATGTACGACCTGGACGGCAATGGCTACATCAGCCGCAGTGAAATGCTGGAGATAGTGCAGGCCATCTACAAGATGGTGTCCTCCGTGATGAAGATGCCTGAGGACGAGTCCACGCCCGAAAAGCGAACAGACAAGATCTTCAGACAGATGGACACAAACAATGATGGCAAACTGTCCCTGGAAGAATTCATCAAAGGTGCCAAGAGCGACCCATCCATTGTCCGGTTGCTGCAGTGTGATCCCAGCAGTGCTAGCCAGTTC |

**Table S2 The list of primers used for RT-qPCR.**

| **Name** | **Primer Sequence** | |
| --- | --- | --- |
| H-VILIP3 | Forward | 5’- GATGGTGTCGTCTGTGATGAAG-3’ |
|  | Reverse | 5’- TCATTGTTGGTGTCCATCTGC-3’ |
| H-β-actin | Forward | 5’- CATGTACGTTGCTATCCAGGC-3’ |
|  | Reverse | 5’- CTCCTTAATGTCACGCACGAT-3’ |
| H-GAPDH | Forward | 5’- GCACCGTCAAGGCTGAGAAC-3’ |
|  | Reverse | 5’- TGGTGAAGACGCCAGTGGA-3’ |
| H-GCLC | Forward | 5’- TCTCCAGGTGACATTCCAAGC-3’ |
|  | Reverse | 5’- ACTCCCCAGCGACAATCAAT-3’ |
| H-NQO1 | Forward | 5’- TGGAAGGATGGAAGAAACGC-3’ |
|  | Reverse | 5’- TGGTTGTCAGTTGGGATGGAC-3’ |
| H-HO-1 | Forward | 5’- TGCTGACCCATGACACCAAG-3’ |
|  | Reverse | 5’- GGGCAGAATCTTGCACTTTGTT-3’ |
| M-VILIP3 | Forward | 5’- TGGGCCTTTAGCATGTACGA-3’ |
|  | Reverse | 5’- TCGCTCTTGGCACCTTTGAT-3’ |
| M-β-actin | Forward | 5’- GGCTGTATTCCCCTCCATCG-3’ |
|  | Reverse | 5’- CCAGTTGGTAACAATGCCATGT-3’ |
| M-GAPDH | Forward | 5’- TGTCTCCTGCGACTTCAACA-3’ |
|  | Reverse | 5’- GGTGGTCCAGGGTTTCTTACT-3’ |
| M-GCLC | Forward | 5’- GATGGAGAGTAGAGTTCCGACC-3’ |
|  | Reverse | 5’- CTTGGACAGCGGAATGAGGA-3’ |
| M-NQO1 | Forward | 5’- GCGAGAAGAGCCCTGATTGT-3’ |
|  | Reverse | 5’- TCGAGTCCTTCAGCTCACCT-3’ |
| M-HO-1 | Forward | 5’- GACAGCCCCACCAAGTTCAA-3’ |
|  | Reverse | 5’- AGCTCCTCAAACAGCTCAATGT-3’ |

**Table S3 The list of indicated antibodies.**

| **Antibody** | **Cat No.** | **Manufacturer** |
| --- | --- | --- |
| VILIP3 | 10989-1-AP | Proteintech |
| GAPDH | 10494-1-AP | Proteintech |
| β-actin | AC026 | ABclonal |
| BACE1 | ab108394 | Abcam |
| PSD95 | A0131 | ABclonal |
| Synaptophysin | A19122 | ABclonal |
| SNAP25 | 14903-1-AP | Proteintech |
| β-Amyloid | 8243T | Cell Signaling Technology |
| β-Amyloid | 803004 | BioLegend |
| Bcl-2 | ab196495 | Abcam |
| Bax | ab32503 | Abcam |
| Cleaved Caspase-3 | 9664T | Cell Signaling Technology |
| Nrf2 | Ab62352 | Abcam |
| Nrf2 | 16396-1-AP | Proteintech |
| Lamin B1 | AB0054 | Abways |
| GCLC | Ab207777 | Abcam |
| NQO1 | 11451-1-AP | Proteintech |
| HO-1 | 10701-1-AP | Proteintech |
| DDDDK-Tag | AE063 | ABclonal |
| PS1 | ab76083 | Abcam |
